# Potentiometric MRI of a Superconcentrated Lithium Electrolyte: Testing the Irreversible Thermodynamics Approach

**DOI:** 10.1021/acsenergylett.1c01213

**Published:** 2021-08-15

**Authors:** Andrew A. Wang, Anna B. Gunnarsdóttir, Jack Fawdon, Mauro Pasta, Clare P. Grey, Charles W. Monroe

**Affiliations:** † Department of Engineering Science, University of Oxford, Oxford OX1 3PJ, U.K.; ‡ The Faraday Institution, Harwell Campus, Didcot OX11 0RA, U.K.; § Department of Chemistry, University of Cambridge, Cambridge CB2 1EW, U.K.; ∥ Department of Materials Science, University of Oxford, Oxford OX1 3PH, U.K.

## Abstract

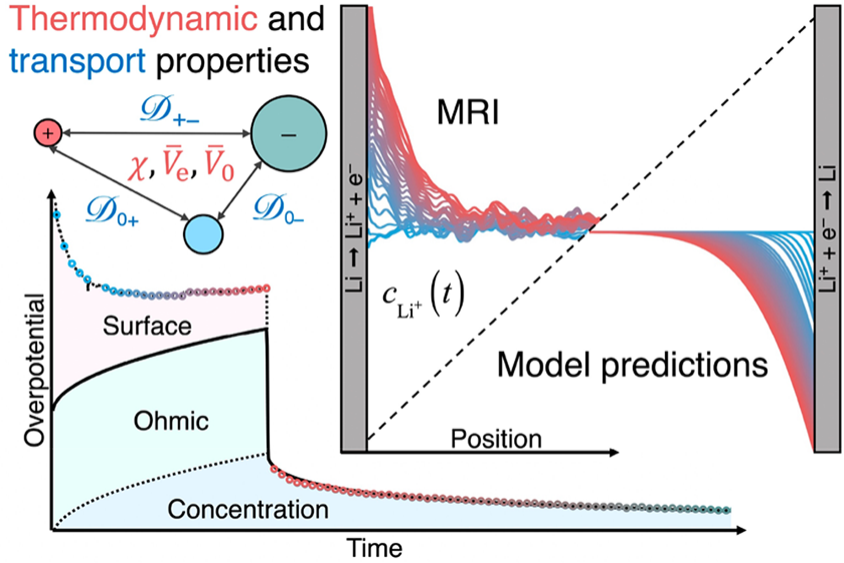

Superconcentrated electrolytes, being highly thermodynamically nonideal, provide a stringent proving ground for continuum transport theories. Herein, we test an ostensibly complete model of LiPF_6_ in ethyl-methyl carbonate (EMC) based on the Onsager–Stefan–Maxwell theory from irreversible thermodynamics. We perform synchronous magnetic resonance imaging (MRI) and chronopotentiometry to examine how superconcentrated LiPF_6_:EMC responds to galvanostatic polarization and open-circuit relaxation. We simulate this experiment using an independently parametrized model with six composition-dependent electrolyte properties, quantified up to saturation. Spectroscopy reveals increasing ion association and solvent coordination with salt concentration. The potentiometric MRI data agree closely with the predicted ion distributions and overpotentials, providing a completely independent validation of the theory. Superconcentrated electrolytes exhibit strong cation–anion interactions and extreme solute-volume effects that mimic elevated lithium transference. Our simulations allow surface overpotentials to be extracted from cell-voltage data to track lithium interfaces. Potentiometric MRI is a powerful tool to illuminate electrolytic transport phenomena.

A spatiotemporal understanding of how chemical species and potential are distributed—on both microscopic and mesoscopic scales—is vital to lithium-battery development. As lithium batteries proliferate across more demanding applications, a strong grasp of electrolytic thermodynamics and mass-transport phenomena will be critical to the optimization of existing technologies and the design of new formulations with advanced capabilities.^1−4^

Local concentration and potential within electrolytic solutions must be managed during battery operation, because their distributions affect several distinct phenomena across a wide range of systems. Polarization of the composition and voltage within a cell as it cycles leads to capacity underutilization and also restricts fast-charging efficiency.^5,6^ Concentration overpotentials are particularly important during operation at high current densities, where they can obfuscate operational limits based on the equilibrium potentials for lithiating graphitic anodes, ultimately leading to lithium-metal plating.^7,8^ The dendritic morphology of plated lithium, a key barrier to lithium-metal batteries, is strongly affected by the instantaneous liquid-phase composition and voltage near the electrode surfaces, as well as by additives that alter solution thermodynamics.^9−13^ Both solid–electrolyte interphase (SEI) formation and degradation side reactions are governed by reaction processes whose rates depend on interfacial electrolyte concentrations.^14−16^ On a more fundamental level, studies of electrode kinetics based on cell voltage require true surface overpotentials to be distinguished from potential drops away from the interfaces, such as Ohmic and diffusive losses.^17^

The properties of liquid electrolytes depend strongly on their salt content. Renewed attention has been given to superconcentrated electrolytes, which in some cases exhibit widened voltage stability windows and higher apparent rate capabilities.^18−20^ In the superconcentrated regime, cation, anion, and solvent speciation is incredibly complex, dominated by coordinated aggregation phenomena such as solvent binding and ion pairing.^4,21−24^ Because many studies of superconcentrated electrolytes do not include complete assessments of transport and thermodynamic properties, the literature remains unclear about whether the advantages stem from interfacial or bulk characteristics.^25−27^ It is therefore of interest to examine how concentration and potential polarization evolve within superconcentrated electrolytes subjected to applied currents.

Newman’s concentrated-solution theory derives from the Onsager–Stefan–Maxwell (OSM) model of multicomponent diffusion—an implementation of irreversible thermodynamics that takes electrochemical-potential gradients, rather than concentration gradients, to be the fundamental forces that drive diffusion.^28^ Unlike Nernst–Planck dilute-solution theory, OSM theory also accounts explicitly for ion/ion diffusional drag interactions, which generally cannot be neglected in battery electrolytes.^29,30^ The thermodynamic principles that underpin OSM theory also support the consistent inclusion of standard equilibrium properties, such as component partial molar volumes and Darken thermodynamic factors.^31,32^

For binary electrolytic solutions wherein speciation kinetics is very fast, a single (Darken) thermodynamic factor χ accounts for deviation from the ideal Nernstian relationship between concentration polarization and concentration overpotential. Also, effective electrolyte and solvent partial molar volumes, denoted *V̅*_e_ and *V̅*_0_, respectively, parametrize the concentration dependence of the solution’s density. Together these three thermodynamic properties should provide a complete description of an equilibrated electrolyte at constant temperature *T* and pressure *p*. With the thermodynamic parameters in hand, the three Stefan–Maxwell diffusivities describing pairwise species/species diffusional-drag interactions map into an ionic conductivity κ, salt diffusion coefficient *D*, and cation transference number relative to the solvent velocity *t*_+_^0^. Thermodynamic constraints demand that these six essential material properties vary only with local salt molarity *c* at fixed *T* and *p*.

When applied to a three-species electrolytic solution,^91^ OSM theory produces two independent force-explicit transport equations that describe the dynamical state.^28^ Inversion of these equations leads to two flux-explicit transport laws, parametrized by the six key composition-dependent properties.^33−35^ First, the total molar cation flux *N⃗*_+_ is described in terms of contributions from diffusion, migration driven by the ionic current density *i⃗*, and convection at the volume-average velocity *v⃗*^□^ (defined in the Supporting Information, eq S4) as
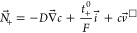
1where *F* represents Faraday’s constant. Second, a modified form of Ohm’s law breaks the current density down into an Ohmic contribution, driven by the local potential gradient, and a Nernstian contribution, driven by gradients in composition:
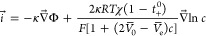
2in which *R* is the gas constant and *T* is the absolute temperature. To produce the simulations below, eqs 1 and 2 were augmented by local state equations, material balances, and boundary conditions, which are detailed in section S2.A of the Supporting Information.

Several attempts to parametrize the composition dependences of all six key properties in eqs 1 and 2 have been made, involving a range of experimental and numerical approaches.^36−43^ The convection term in eq 1 is typically disregarded, but volume-related transport phenomena such as Faradaic convection may become dominant in electrolytes where the salt fraction is high.^44−46^ Only a few groups have attempted to validate measured parameters, by comparison of theoretical predictions to voltage measurements or otherwise.^36−39^

In situ methods such as nuclear magnetic resonance imaging (MRI), X-ray scattering, and confocal Raman spectroscopy have been employed to visualize the time evolution of electrolyte-concentration profiles.^9,47−49^ In particular, inverse modeling techniques have been applied to MRI data to extract transport parameters.^47,50−52^ Material properties determined in this way are highly sensitive to prior assumptions about the composition dependences of properties, as well as to the model variant that is chosen when fitting the data. Most inverse modeling of MRI has used models reliant on several restrictive assumptions, e.g., dilute-solution theory,^53^ modeling without independent consideration of the thermodynamic factor,^47^ or neglect of solute-volume effects,^54^ or has employed *ad hoc* adaptations to account for additional phenomena such as dendrite growth.^53,55,56^ Most recently, Bazak et al. extracted composition-dependent transference numbers and diffusivities from steady-state concentration profiles measured with MRI by inverse modeling based on a concentrated-solution theory that excluded the thermodynamic factor χ and the Faradaic-convection phenomenon.^50^ No MRI studies have validated the properties extracted from concentration measurements against the cell voltage, which should be wholly determined by the transport model during open-circuit relaxations.^57^

In situ approaches to date have focused on parameter estimation, but a more foundational question remains: Is the concentrated-solution transport theory itself valid? That is, does an ostensibly “complete” transport model based on irreversible thermodynamics, fully parametrized ex situ in the traditional, circumstantial way, in fact predict the concentration profiles and transient cell voltages seen in situ?

To address this question, we report a complete set of thermodynamic and transport properties for solutions of lithium hexafluorophosphate (LiPF_6_) in ethyl methyl carbonate (EMC) across the entire solubility range, including the superconcentrated regime near the salt’s saturation limit. The LiPF_6_:EMC system is representative of superconcentrated electrolytes based on linear-carbonate solvents.^18^ Highly nonideal speciation in the superconcentrated regime is confirmed by observations with Raman spectroscopy. Structural observations derived from the Raman spectra correlate well with trends in the partial molar volumes, confirming that the inclusion of solute-volume phenomena within the OSM model of the bulk electrolyte is critical to predict ion fluxes accurately at high salt concentrations.^36,37,44^ The transport model is parametrized using a bespoke suite of electrochemical measurements designed to isolate the concentration dependences of individual properties.^36^ Finally, potentiometric MRI data gathered during galvanostatic polarization/relaxation demonstrate strong agreement with transient concentration and voltage profiles predicted by model simulations. These results confirm the high fidelity and microscopic predictive capability of the solution-phase transport model, justifying its use to isolate the voltage signatures of interfacial phenomena.

## Species Interactions Increase with Salt Molarity

**Figure 1:**
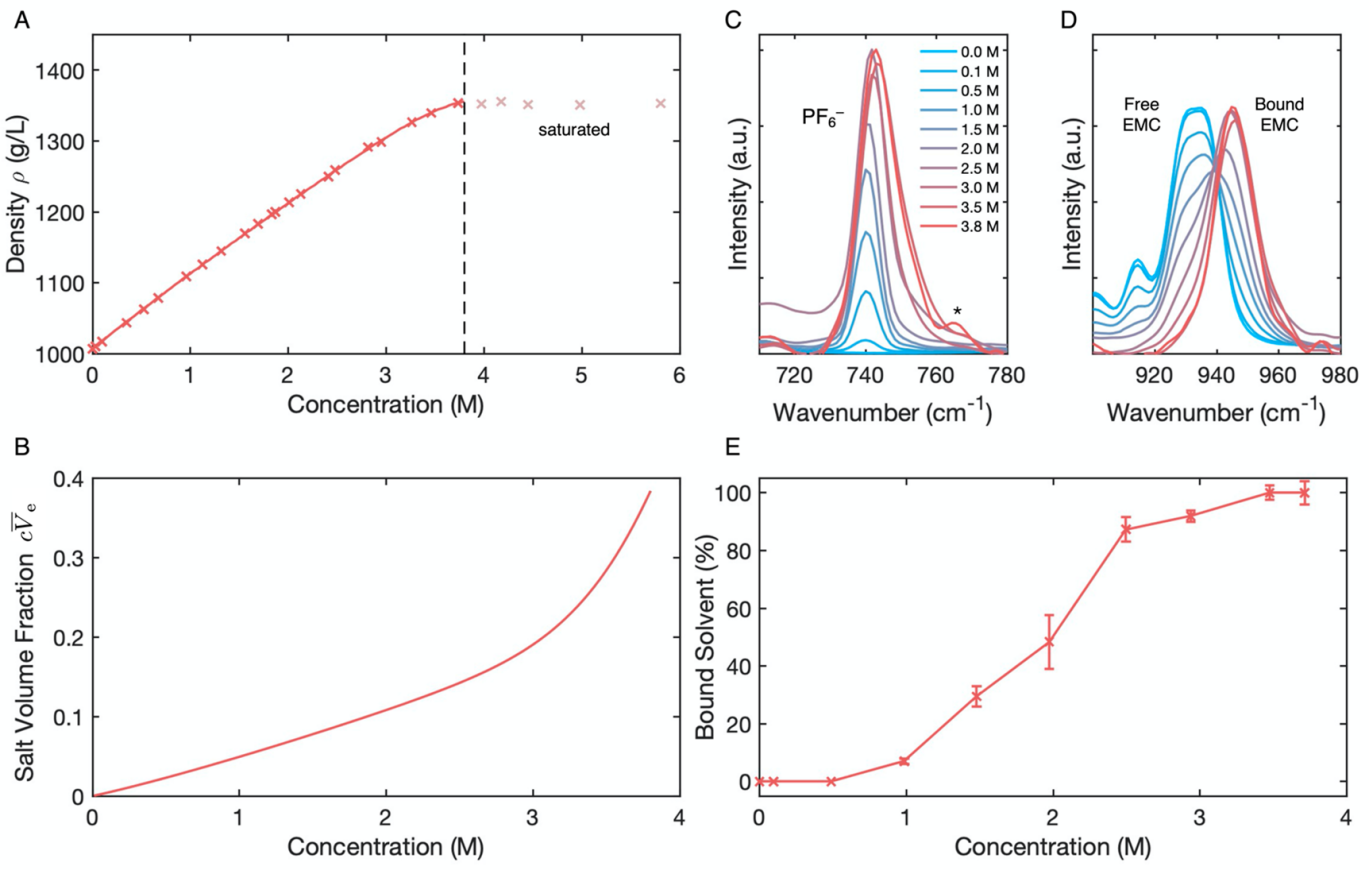
Variation of physicochemical properties and molecular interactions with salt concentration from 0 M to saturation (3.8 M). (A) Density measurements for LiPF_6_:EMC solutions at 25 °C. The dashed line (- - -) at 3.8 M demarcates saturation. (B) Salt volume fraction, derived from salt molarity *c* and solute partial molar volume *V̅*_e_ computed with Supporting Information eq S14). (C) Raman spectra for LiPF_6_:EMC in the range of 720–780 cm^–1^; the asterisk indicates crystalline LiPF_6_. (D) Raman spectra for LiPF_6_:EMC in the range of 920–980 cm^–1^ showing solvent interaction. (E) Bound solvent fraction, as calculated by comparing the change in peak fit areas between the neat EMC solvent and saturated LiPF_6_:EMC solution. Error bars represent uncertainty due to peak fitting of the EMC C–O stretching band (see the Supporting Information for further details)

Figure 1 presents physicochemical data and Raman spectra for LiPF_6_:EMC solutions as functions of their superficial salt molarity *c*. As the saturation limit of LiPF_6_:EMC at *c* = 3.8 M is approached, the solution’s mass density ρ, shown in Figure 1A, levels off. (These data were gathered from filtered supernatant liquid, so the horizontal slope above 3.8 M reflects that all the added salt has not dissolved.) The change in the density’s slope with respect to molarity, from 85 g/mol at 3 M to 0 g/mol beyond the saturation limit of 3.8 M, indicates a large increase in the salt partial molar volume *V̅*_e_, as described in the Supporting Information (eq S14). Figure 1B shows that the salt’s volume fraction within solution rises sharply near saturation, reaching nearly 40% at 3.8 M. This suggests that significant Faradaic convection (bulk flow driven by the volume flux of Li^+^ across electrode boundaries) should accompany interfacial redox reactions in concentrated electrolytes.^44^

Changes in the Raman bands associated with PF_6_^–^ and EMC, shown in panels C and D of Figure 1, respectively, indicate dramatic changes in solution structure as salt content rises. Figure 1C shows increased shift of the PF_6_^–^ stretching vibration from its expected 741 cm^–1^ band and an increase in intensity as molarity rises, trends which have been correlated with a rising extent of ion association.^58−60^ Data from Figure 1D was used to assess the coordination of solvent with lithium cations by comparing the integrated peak fit area around ∼930 cm^–1^, associated with asymmetric C–O stretching of the free molecule, with that around ∼946 cm^–1^ associated with Li^+^–EMC coordination.^61,62^ There is also a peak at ∼918 cm^–1^, which has been attributed to the stretching of C=O double bonds;^63^ in unbound EMC its height correlates with the peak height at ∼930 cm^–1^. Figure 1E presents how the percentage of bound solvent, computed using these peak areas, varies with molarity. Below 1 M—the concentration regime for standard lithium-ion-battery electrolytes—our Raman analysis indicates that the majority of EMC moves freely, but the opposite holds above 3 M, where more than 90% of the EMC is coordinated with lithium. The monotonic increase in solvent coordination with concentration qualitatively agrees with prior studies of LiPF_6_ in linear-carbonate solvents,^64^ although infrared spectroscopy tends to show higher coordination at lower concentrations.^65^

A relatively steady transition to a state wherein ions and solvent are highly coordinated occurs across the 1–3 M range, commensurate with behavior observed for other nonaqueous solutions.^4^ As such, theories that assume infinite dilution or neglect ion–ion interactions cannot account entirely for transport at high concentrations, which is driven by both the typical “vehicular” transport mode, in which solvated ions move freely through the solution, as well as a “structural” mode, in which cations move by rearranging the bonds in their local coordination networks.^29,66^ Dilute-solution theory and many popular concentrated-solution models also neglect a “kinematic” transport mode, in which the bulk electrolyte is driven to move because salt flux carries a portion of the solution volume along with it. (Faradaic convection is an example of a kinematic transport mode.) Consistent models must include both species–species interactions and solute-volume effects.^36,37,44^

## Ex Situ Property Measurements

**Figure 2:**
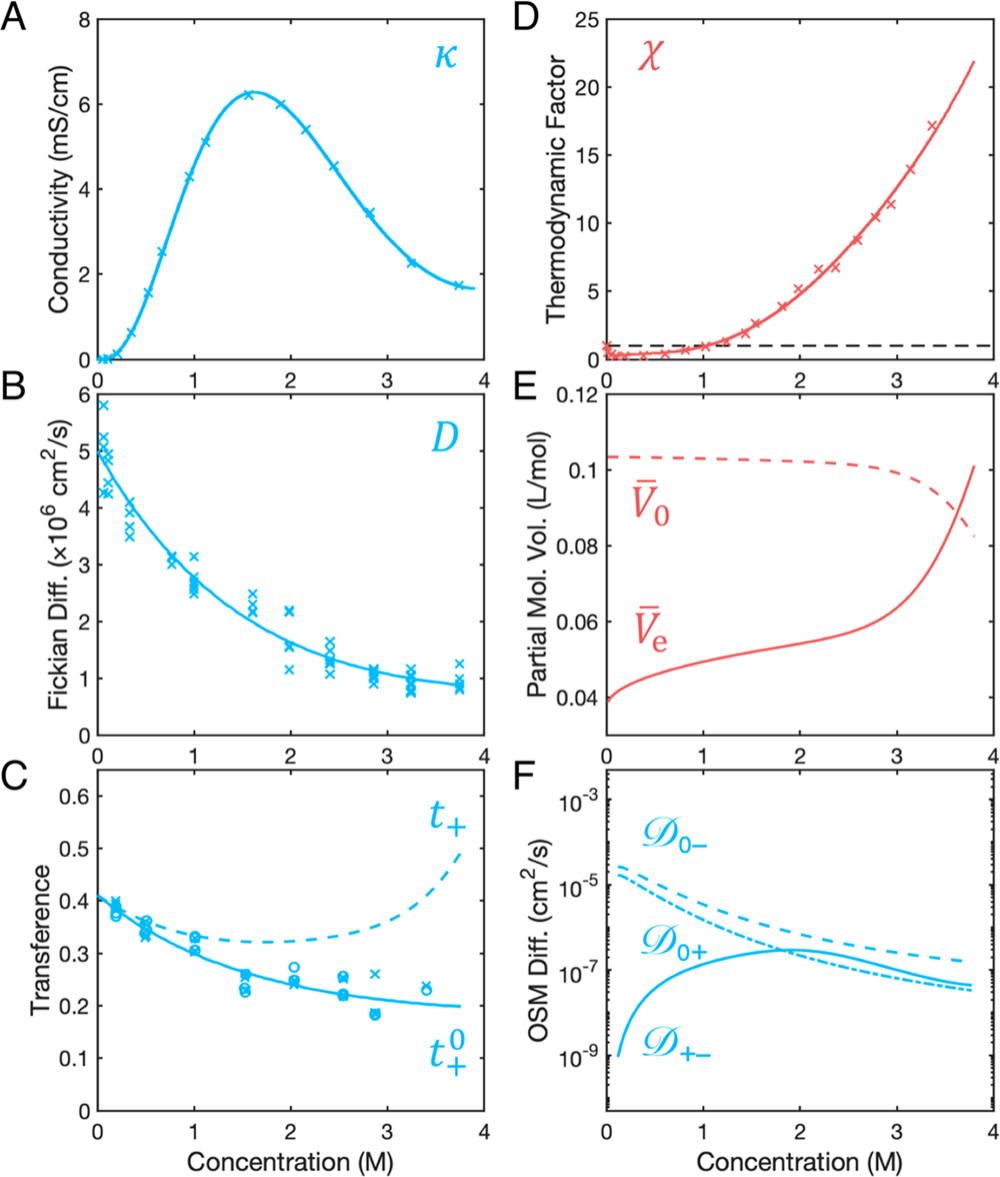
Transport (blue) and thermodynamic (red) properties for LiPF_6_:EMC measured at 25 °C. Composition-dependent correlation curves with confidence intervals and tables of the raw experimental data are provided in Tables S1 and S2. (A) Ionic conductivity κ as measured by AC conductometry. (B) Salt diffusivity *D* as measured by potentiometric restricted diffusion. (C) Transference number *t*_+_^0^ (—) as measured by Hittorf experiments, determined from the cathodic (○) and anodic (×) chambers of the Hittorf cell as depicted in Figure S2, alongside the transport number *t*_+_ (- - -) that would arise from data processing without Faradaic convection. (D) Darken thermodynamic factor χ as measured by shifting-reference concentration cells. The dashed line shows the ideal value of unity. (E) Partial molar volumes for solvent *V̅*_0_ (- - -) and salt *V̅*_e_ (—) from densitometry (Supporting Information eqs S13 and S14). (F) Onsager–Stefan–Maxwell diffusion coefficients between EMC and PF_6_^–^_0–_ (- - -), EMC and Li^+^_0+_ (·–·), and Li^+^ and PF_6_^–^_+–_ (—), determined from properties presented in panels A–E (see the Supporting Information, section S.2D)

Prior measurements of transport and thermodynamic properties for LiPF_6_:EMC up to 2 M were extended into the superconcentrated regime using the experimental characterization suite developed by Wang et al.^36^ The composition dependence of every material parameter involved in eqs 1 and 2 is presented in Figure 2. Ionic conductivity κ (Figure 2A) trends through a maximum at ∼1.5 M, as the entropic driving force for salt dissociation at high dilution is counterbalanced by the increased diffusional drag that ions experience at higher concentrations. Salt diffusivity *D* (Figure 2B) falls monotonically with concentration, consistent with a decrease in both ions’ mobilities relative to the solvent. The Hittorf transference number *t*_+_^0^ (Figure 2C) trends downward, showing that Li^+^ carries a decreasing fraction of the ionic current as salt content rises. Note that this observation has been reported before^26^ and opposes the conclusion drawn by other groups that lithium transference appears higher in the superconcentrated regime.^19,67^ The discrepancy can be explained by a difference in definitions between the Hittorf transference number *t*_+_^0^ and the transport number *t*_+_, which is equivalent to the transference parameter extracted by the Bruce–Vincent method.^19,37,67,68^ Whereas *t*_+_^0^ is defined within concentrated-solution theory to ensure that it is a truly isolable property of the bulk electrolyte,^36^ the formulas used to quantify *t*_+_ derive from Nernst–Planck dilute-solution theory, thereby neglecting both ion–ion diffusional interactions^69^ and convective effects^35^ in the data processing. When the volume fraction of salt in a solution is appreciable, the solution-volume change that accompanies interfacial reactions drives a bulk flow, which elevates the apparent transport number *t*_+_(36,44) through convection—an effect that has nothing to do with bulk transference.^41^ Thus, the “enhanced transference” of superconcentrated electrolytes seen by prior researchers may more accurately be interpreted as enhanced Faradaic convection owing to the high volume fraction of salt.

The thermodynamic properties shed light on solution behavior in the superconcentrated regime as well. At moderate concentrations, up to 1 M, poor salt dissociation suppresses χ below its ideal value of unity, as a consequence of EMC’s low relative permittivity (approximately 3).^3,36,70^ Solution nonidealities grow at concentrations above 1 M, driving a dramatic increase in χ (Figure 2D). In line with the Raman spectra presented in Figure 1, the rise in salt activity (Supporting Information eq S17) likely is due to an ever-increasing extent of solute–solvent coordination.^70^ Note also that changes in the thermodynamic factor ultimately reflect changes in the salt’s chemical potential. This interpretation of the thermodynamic factor is also consistent with the trends seen in the partial molar volumes (Figure 2E): the electrolyte’s partial molar volume rises steadily, with a steeper change above 3 M. Salt formula units in solution would be expected to attain elevated effective volumes if more solvent molecules become coordinated with them. Thus, these extreme nonideal variations in thermodynamic behavior would be expected to impact the thermodynamics of solvation/desolvation reactions at electrode surfaces.

Finally, OSM diffusivities, shown in Figure 2F, express the mobility of one species relative to another. Casting these data in terms of Onsager drag coefficients (Figure S3.A), rather than diffusivities, also corroborates the observations that solute–solvent drag interactions increase steadily as the concentration tends toward saturation, that significant cation–anion coordination impacts the ionic conductivity nonmonotonically with concentration, and that all three pairwise diffusional drag interactions (cation–solvent, anion–solvent, and cation–anion) have similar magnitudes in the superconcentrated regime (Figure S3.B). The Supporting Information (Figure S3C) also presents Onsager diffusivities, which can be interpreted as measures of correlation decay. These reveal that cation/anion fluctuations transition from correlated to anticorrelated at the conductivity maximum.

## Potentiometric MRI

The response of a 3 M LiPF_6_:EMC solution sandwiched between planar lithium-metal electrodes with an 8 mm interelectrode spacing was investigated to probe concordance between potentiometric MRI data and the predictions of irreversible thermodynamics based in the OSM concentrated-solution theory. Experiments were performed using sealed custom Swagelok-style PEEK cells with cylindrical internal geometry (Figure S7). The cells were subjected to galvanostatic pulse–relaxation experiments, in which a 60 μA (0.48 mA/cm^2^) current pulse was applied for 2 h, followed by an open-circuit (0 mA/cm^2^) hold; the cell voltage and MRI signal were tracked during both the pulse and the relaxation. Cells were oriented vertically, with positive current in an upward direction to minimize buoyancy effects (free convection) arising from density gradients that accompany concentration polarization within the cell.^28,47^^19^F MRI concentration profile imaging was performed on the PF_6_^–^ anion, ^19^F representing the strongest signal from all the ions present in this system; Li^+^ concentration was inferred via the local electroneutrality approximation. Image acquisition occurred approximately every 4 min, using a 1D spin–echo pulse sequence with the external magnetic field aligned along the direction of ion transport.^71^ Synchronous cell-voltage data was gathered throughout the duration of the experiment and is discussed below.

Model calculations were performed using COMSOL Multiphysics software; the code used to perform simulations is available on GitHub.^72^ Transient balances of charge, volume, and cation concentration in the axial direction, a coupled partial differential equation system determined by eqs 1 and 2, were solved with appropriate boundary conditions, as detailed in the Supporting Information (section S2.A).

**Figure 3:**
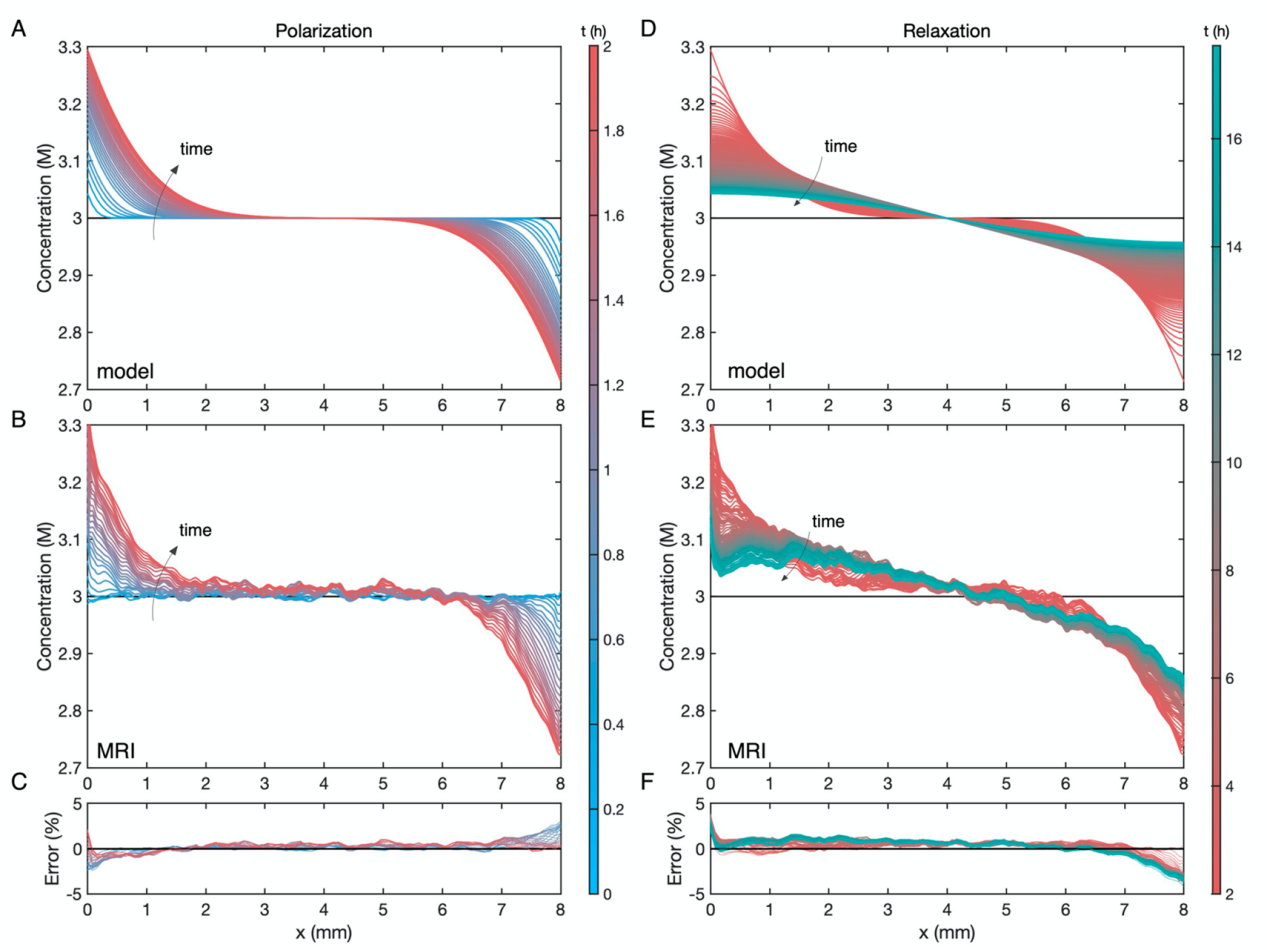
Comparison between dynamic concentration gradients simulated via the ex situ parametrized model and profiles captured by potentiometric ^19^F MRI during galvanostatic polarization/relaxation of 3 M LiPF_6_:EMC between planar Li-metal electrodes at 25 °C. Color varies from steel blue, through maroon, to sea green as time increases. (A) Model-simulated concentration profiles for a 2 h pulse polarization at 0.48 mA/cm^2^. (B) ^19^F MRI concentration profiles measured for a 2 h pulse polarization at 0.48 mA/cm^2^. (C) Percentage error in microscopic concentration between MRI measurements and model predictions during the polarization step. (D) Model-simulated concentration profiles during open-circuit relaxation after the 2 h pulse. (E) ^19^F MRI concentration profiles measured during open-circuit relaxation after the 2 h pulse. (F) Percentage error between MRI measurements and model predictions during the relaxation step.

Simulation output is compared with the experimental data in Figure 3. It should be emphasized that the only parameters input to the model are the electrode spacing, the pulse duration, and the applied current density; no material parameters from the property set in Table S1 were tuned to achieve the agreement between panels A and B or panels D and E of Figure 3. The macroscopic model indeed predicts those microscopic states with good accuracy: panels C and F of Figure 3 demonstrate that the theoretical and experimental concentrations align within ±5% at all times. This validation exercise supports the use of continuum-scale theories based in irreversible thermodynamics to complement the modeling of degradation or formation processes in lithium batteries, which usually involve mechanistic reaction models that incorporate solution-phase species concentrations local to interfaces.^73^

The concentration response during the constant-current pulse, shown in Figure 3A,B, is governed by diffusion, migration, and Faradaic convection (eq 1).^44,74^ The balance of these effects determines the slope of the concentration profile at the electrolyte’s boundaries, which is generally proportional to the applied current. As time passes, diffusion boundary layers penetrate toward the center of the cell, a process dominated by the balance between diffusion, which controls the penetration depth of the layer, and migration, which determines the rate that the excess concentration grows or decreases at the boundaries.

Figure 3E presents the open-circuit concentration relaxation recorded by MRI. Whereas previous in situ MRI characterizations have studied the galvanostatic polarization step exclusively,^47,50−52^ the relaxation data reported here is particularly useful because it is governed by diffusion alone. During the relaxation, the concentration profile becomes flat at the boundaries, confirming the absence of the current-proportional effects of migration and Faradaic convection. Previous analyses of restricted diffusion experiments have relied on alternative single-point or integral-average composition indicators, such as concentration overpotential, ultraviolet–visible spectroscopy, or local conductivity measurements,^33,36,75−77^ which cannot validate details of the microscopic concentration distributions revealed by MRI.

Note that the disagreement between the predicted and simulated concentration responses, quantified in Figure 3C,F, is always largest at the electrolyte’s edges—the spatial domain that is also subject to higher experimental error.^54^ This is due in part to susceptibility effects from the Li metal electrodes. The presence of an electronic conductor commonly causes radio frequency (RF) attenuation or dispersion, which affects the MRI calibration significantly. Still, there is notable additional error on the side of the cell where Li plating occurs (*x* = 8 mm in Figure 3). Over a distance of about 0.5 mm from the edge on the right of Figure 3E, the transient MRI profiles do not appear to relax as fast as the predictions in Figure 3D, but the left sides of the distributions in both panels relax on apparently similar time scales.

It should be emphasized that lithium plating occurred at the right side of the domain shown in panels B and E of Figure 3, whereas lithium was stripped at the left. Changes in lithium surface morphology during plating are well-documented and might be a source of the apparently slower relaxation at the right. Formation of a mossy lithium layer would lower the effective diffusivity observed in regions where lithium was deposited, but this is unlikely given the relatively small amount of lithium plated during the current pulse. A charge density of 0.95 mAh/cm^2^ was passed during the pulse, which would correspond to a dense Li layer 4.7 μm thick. If the mossy lithium were to spread over the distance suggested by the figure, the porosity of plated lithium would have to be more than 99%, which is unlikely in light of the microscopic observations of lithium electrodeposition by, for example, Wood and colleagues.^78^ The volume of lithium plated is far too low to produce structures that isolate pockets of electrolyte. If needle-like lithium filaments were present, it would be possible for them to protrude hundreds of micrometers into the electrolyte. This deposit morphology cannot account for the slow observed concentration relaxation, however; porosities above 99% would have negligible impact on effective diffusivity and therefore cannot explain the apparently slower relaxation.^79,80^ It is much more likely that the anomalous region near the electrode is due to intrinsic error in the MRI measurement. RF shielding and distortion effects are commonly observed in the nuclear magnetic resonance (NMR) of metallic materials. Layers of metal powders, which one would expect are structurally similar to mossy lithium from the electromagnetic standpoint, have been observed to induce inhomogeneities in MRI signals much larger than the layer thickness.^81−83^ It is noteworthy that an enhanced dispersive phenomenon due to the porosity of deposited lithium may be further exacerbated in experiments that rely on longer-duration polarizations (and thus a greater thickness of mossy Li) to achieve steady-state concentration profiles. Past sources from the inverse-modeling literature have typically navigated this issue by excluding data within some distance of the electrode from the data processing.^47,84^

For electrolytes in the superconcentrated regime, consistent multicomponent transport models are necessary to account for the complex interactions present. In particular, because ∼25% of the electrolyte volume is contributed by salt (cf. Figure 1B), there is a substantial Faradaic convection effect, in which the cation exchange owing to plating and stripping of lithium at the solution boundaries induces a volume flux in the bulk whose current dependence differs from that of migration.^36,44^ The neglect of solute-volume phenomena explains why inverse modeling of MRI data has yielded current-density-dependent transference numbers, such as those reported by Bazak et al.^84^ Profiles in Figures 3 and S11 show that the parameter sets in this work, which are taken to depend only on local electrolyte composition, suffice to predict the system response.

## Voltammetric Validation

**Figure 4:**
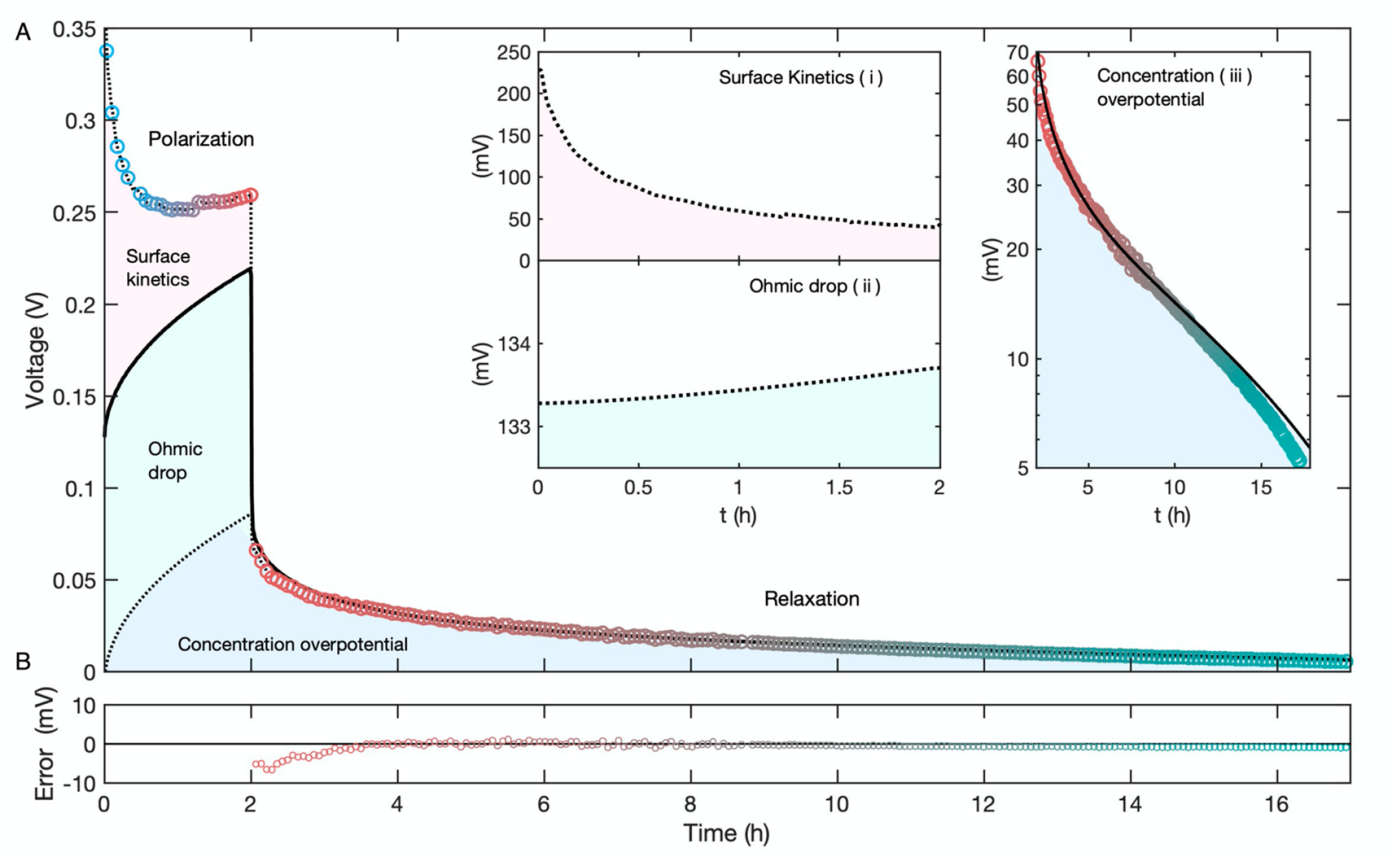
Voltage response of highly concentrated electrolyte during 2 h, 0.48 mA/cm^2^ galvanostatic polarization, and subsequent open-circuit relaxation. (A) Experimental cell voltage recorded during in situ MRI (○); colors vary over time consistently with the legends in Figure 3. COMSOL-simulation predictions of solution-phase overpotentials (solid —) due to Ohmic and Nernstian contributions. Inset A.i: Surface-overpotential contribution to cell voltage (red-shaded area) as inferred from the difference between model-predicted overpotentials and measured voltage response. Inset A.ii: Ohmic drop (green-shaded area) as calculated from simulation output by integration of the corresponding term in eq 2. Inset A.iii: Semilog plot of concentration overpotential (blue-shaded area) showing its transient decay during the relaxation step. (B) Voltage residual between experiment and simulation during the relaxation step.

During a galvanostatic pulse, the measured voltage consists of contributions from Ohmic drop associated with ionic conductivity; concentration overpotential due to the developing concentration difference across the cell; and two surface overpotentials, which drive stripping and plating half-reactions at the Li metal electrodes. The concentrated-solution theory accounts only for the potential drop within the bulk solution phase and therefore models only the first two of these contributions (Figure 4A, solid line).^44^ During the open-circuit hold the surface overpotentials vanish and the model used here should account for all of the cell voltage.

Model prediction matches the observed voltage decay well (within 7 mV at all times in Figure 4B) during the open-circuit relaxation step, confirming the validity of the OSM parameters quantified ex situ. Notably, independent consideration of the composition dependence of the thermodynamic factor, χ, is crucial for translating concentration polarization into accurate concentration overpotentials across the electrolyte. Dilute-solution models based on Nernst–Planck theory require that χ = 1 in eq 2 and therefore have no means of accounting for the extreme nonideality of superconcentrated electrolytes, for which matching the observed diffusion potentials requires χ values of order 10 (cf. Figure 2D) .^36,85^

In battery configurations containing superconcentrated electrolytes, overpotential management is critical to performance optimization. Electrolytes typically permeate porous separators and electrodes in practical cells. Additional mass-transport limitations due to pore geometry generally exacerbate the development of both concentration gradients and overpotentials.^86^ Although shorter interelectrode distances reduce the barriers to high-rate operation by raising the mass-transfer-limited current, physics-based simulations similar to the one deployed here have shown that practical cell geometries are susceptible to severe concentration gradients that can swing from the equilibrium composition by ±100% under reasonable power loads.^87^

The close agreement between the experimental and predicted voltages during open-circuit relaxation justifies further processing of the voltages observed during the current pulse. By ascribing the difference between observation and prediction to surface overpotentials, quantitative insight can be gained about the redox kinetics of Li-metal electrodes immersed in superconcentrated electrolytes. The nonmonotonic voltage response to applied current has been attributed to surface-morphology changes of lithium electrodes by Wood and colleagues.^78,79^Figure 4A(i) shows that when concentration overpotentials and bulk Ohmic losses are corrected out of the data, the net surface overpotential in fact decreases monotonically. Note that this surface overpotential encapsulates all interfacial phenomena, for example, lithium plating/stripping kinetics and Ohmic resistance of the SEI. The accuracy of the MRI concentration profiles further allows this surface overpotential to be analyzed in terms of two composition-dependent Butler–Volmer kinetic mechanisms in series.^88^ A fitting procedure based on a simple Butler–Volmer kinetic model (Supporting Information, section S2.G) produces an effective exchange-current density that increases linearly with respect to the square root of time, traditionally consistent with a diffusion-limited surface roughening process during Li deposition.^78,89,90^ This overall trend suggests that for the galvanostatic polarization experiments presented, the increase in Li surface area (and its accompanying SEI) outcompetes any increase in interfacial resistance from SEI thickening elsewhere. It is difficult to construct reference-electrode configurations that offer position-dependent information within an electrochemical cell;^40^ complete transport and thermodynamic descriptions of bulk electrolytes could enable further quantitative analysis of interfacial processes.

In conclusion, superconcentrated electrolytes have distinctive structures, evidenced by the partial molar volumes of their components, their extreme thermodynamic nonideality, and their high degree of solvent coordination. Within LiPF_6_:EMC, more than 25% of the solution volume is carried by salt in the superconcentrated regime above 3 M, and concentration overpotentials are amplified by a factor of 10 or more. The OSM concentrated-solution theory accounts for all the quasi-equilibrated pairwise species interactions that occur locally within this system. This study reveals that the theory remains suitable in the superconcentrated regime, so long as the thermodynamic factor and Faradaic convective effects are included.

A systematic parametrization of transport and thermodynamic properties for LiPF_6_:EMC was performed using a suite of independent experiments across the solution’s solubility range. Significantly, instead of applying an inverse-modeling approach, we instead validated the theoretical framework by comparing model predictions to independently gathered potentiometric MRI data. We found that local states within an electrolyte pulsed with a constant current are predicted well by a model whose parameters are circumstantially inferred by a set of independent experiments that record global system characteristics. For superconcentrated LiPF_6_:EMC, artificially higher lithium transference is more accurately attributed to a solute-volume effect. By exploiting open-circuit relaxations, we also eliminated the voltage signatures of phenomena that are not described by the solution-phase transport model, such as interfacial reactions.

This work confirms that models based in irreversible thermodynamics provide accurate predictions of local states within electrolytes. Theories of this type allow ready extension to include thermal and viscous properties, which enables rigorous modeling beyond the isothermal and isobaric scenarios assumed by most continuum electrolyte models in use today. Appropriately parametrized simulations like those described here could be combined with potentiometric MRI in the future to study polarization limitations in demanding applications, manage composition-dependent degradation reactions, and infer morphological evolution of Li-metal interfaces to unlock the performance of next-generation batteries.

## Supporting Information Available

Experimental procedures for transport and thermodynamic property characterization, Raman spectroscopy, and potentiometric MRI (Section S1); model formulation and transport parameters, as well as the raw data obtained when measuring the model parameters, full Raman spectra, additional in situ MRI data, and an analysis of interfacial kinetics (Section S2) (PDF)

## References

[ref1] Logan E. R.; Dahn J. R. Electrolyte Design for Fast-Charging Li-Ion Batteries. Trends Chem. 2020, 2 (4), 354–366. 10.1016/j.trechm.2020.01.011.

[ref2] Amanchukwu C. V. The Electrolyte Frontier : A Manifesto. Joule 2020, 4 (2), 281–285. 10.1016/j.joule.2019.12.009.

[ref3] Xu K. Nonaqueous Liquid Electrolytes for Lithium-Based Rechargeable Batteries. Chem. Rev. 2004, 104 (10), 4303–4417. 10.1021/cr030203g. 15669157

[ref4] Borodin O.; Self J.; Persson K. A.; Wang C.; Xu K. Uncharted Waters: Super-Concentrated Electrolytes. Joule 2020, 4 (1), 69–100. 10.1016/j.joule.2019.12.007.

[ref5] Lain M. J.; Kendrick E. Understanding the Limitations of Lithium Ion Batteries at High Rates. J. Power Sources 2021, 493, 229690 10.1016/j.jpowsour.2021.229690.

[ref6] Kasnatscheew J.; Rodehorst U.; Streipert B.; Wiemers-Meyer S.; Jakelski R.; Wagner R.; Laskovic I. C.; Winter M. Learning from Overpotentials in Lithium Ion Batteries: A Case Study on the LiNi_1/3_Co_1/3_Mn_1/3_O_2_ (NCM) Cathode. J. Electrochem. Soc. 2016, 163 (14), A2943–A2950. 10.1149/2.0461614jes.

[ref7] Arora P.; Doyle M.; White R. E. Mathematical Modeling of the Lithium Deposition Overcharge Reaction in Lithium-Ion Batteries Using Carbon-Based Negative Electrodes. J. Electrochem. Soc. 1999, 146 (10), 3543–3553. 10.1149/1.1392512.

[ref8] Perkins R. D.; Randall A. V.; Zhang X.; Plett G. L. Controls Oriented Reduced Order Modeling of Lithium Deposition on Overcharge. J. Power Sources 2012, 209, 318–325. 10.1016/j.jpowsour.2012.03.003.

[ref9] Chang H. J.; Ilott A. J.; Trease N. M.; Mohammadi M.; Jerschow A.; Grey C. P. Correlating Microstructural Lithium Metal Growth with Electrolyte Salt Depletion in Lithium Batteries Using ^7^Li MRI. J. Am. Chem. Soc. 2015, 137 (48), 15209–15216. 10.1021/jacs.5b09385. 26524078

[ref10] Gunnarsdóttir A. B.; Vema S.; Menkin S.; Marbella L. E.; Grey C. P. Investigating the Effect of a Fluoroethylene Carbonate Additive on Lithium Deposition and the Solid Electrolyte Interphase in Lithium Metal Batteries Using: In Situ NMR Spectroscopy. J. Mater. Chem. A 2020, 8 (30), 14975–14992. 10.1039/D0TA05652A.

[ref11] Gunnarsdóttir A. B.; Amanchukwu C. V.; Menkin S.; Grey C. P. Noninvasive in Situ NMR Study of “Dead Lithium” Formation and Lithium Corrosion in Full-Cell Lithium Metal Batteries. J. Am. Chem. Soc. 2020, 142 (49), 20814–20827. 10.1021/jacs.0c10258. 33226793 PMC7729915

[ref12] Boyle D. T.; Huang W.; Wang H.; Li Y.; Chen H.; Yu Z.; Zhang W.; Bao Z.; Cui Y. Corrosion of Lithium Metal Anodes during Calendar Ageing and Its Microscopic Origins. Nat. Energy 2021, 6, 487 10.1038/s41560-021-00787-9.

[ref13] Louli A. J.; Eldesoky A.; Weber R.; Genovese M.; Coon M.; deGooyer J.; Deng Z.; White R. T.; Lee J.; Rodgers T.; Petibon R.; Hy S.; Cheng S. J. H.; Dahn J. R. Diagnosing and Correcting Anode-Free Cell Failure via Electrolyte and Morphological Analysis. Nat. Energy 2020, 5 (9), 693–702. 10.1038/s41560-020-0668-8.

[ref14] Hall D. S.; Li J.; Lin K.; Stakheiko N.; Baltazar J.; Dahn J. R. A Tale of Two Additives: Effects of Glutaric and Citraconic Anhydrides on Lithium-Ion Cell Performance. J. Electrochem. Soc. 2019, 166 (4), A793–A801. 10.1149/2.1251904jes.

[ref15] Delacourt C. Modeling Li-Ion Batteries with Electrolyte Additives or Contaminants. J. Electrochem. Soc. 2013, 160 (11), A1997–A2004. 10.1149/2.033311jes.

[ref16] Liu Q. Q.; Petibon R.; Du C. Y.; Dahn J. R. Effects of Electrolyte Additives and Solvents on Unwanted Lithium Plating in Lithium-Ion Cells. J. Electrochem. Soc. 2017, 164 (6), A1173–A1183. 10.1149/2.1081706jes.

[ref17] Dickinson E. J. F.; Wain A. J. The Butler-Volmer Equation in Electrochemical Theory: Origins, Value, and Practical Application. J. Electroanal. Chem. 2020, 872, 114145 10.1016/j.jelechem.2020.114145.

[ref18] Cao Z.; Hashinokuchi M.; Doi T.; Inaba M. Improved Cycle Performance of LiNi_0.8_ Co_0.1_Mn_0.1_O_2_ Positive Electrode Material in Highly Concentrated LiBF_4_ /DMC. J. Electrochem. Soc. 2019, 166 (2), A82–A88. 10.1149/2.0291902jes.

[ref19] Suo L.; Hu Y. S.; Li H.; Armand M.; Chen L. A New Class of Solvent-in-Salt Electrolyte for High-Energy Rechargeable Metallic Lithium Batteries. Nat. Commun. 2013, 4, 1–9. 10.1038/ncomms2513. 23403582

[ref20] Peng Z.; Cao X.; Gao P.; Jia H.; Ren X.; Roy S.; Li Z.; Zhu Y.; Xie W.; Liu D.; Li Q.; Wang D.; Xu W.; Zhang J. G. High-Power Lithium Metal Batteries Enabled by High-Concentration Acetonitrile-Based Electrolytes with Vinylene Carbonate Additive. Adv. Funct. Mater. 2020, 30, 2001285–2001285. 10.1002/adfm.202001285.

[ref21] McEldrew M.; Goodwin Z. A. H.; Bi S.; Bazant M. Z.; Kornyshev A. A. Theory of Ion Aggregation and Gelation in Super-Concentrated Electrolytes. J. Chem. Phys. 2020, 152 (23), 234506 10.1063/5.0006197. 32571055

[ref22] Self J.; Fong K. D.; Persson K. A. Transport in Superconcentrated LiPF_6_ and LiBF_4_/Propylene Carbonate Electrolytes. ACS Energy Lett. 2019, 4 (12), 2843–2849. 10.1021/acsenergylett.9b02118.

[ref23] Flores E.; Åvall G.; Jeschke S.; Johansson P. Solvation Structure in Dilute to Highly Concentrated Electrolytes for Lithium-Ion and Sodium-Ion Batteries. Electrochim. Acta 2017, 233, 134–141. 10.1016/j.electacta.2017.03.031.

[ref24] Schammer M.; Horstmann B.; Latz A. Theory of Transport in Highly Concentrated Electrolytes. J. Electrochem. Soc. 2021, 168 (2), 026511 10.1149/1945-7111/abdddf.

[ref25] Suo L.; Zheng F.; Hu Y. S.; Chen L. FT-Raman Spectroscopy Study of Solvent-in-Salt Electrolytes. Chin. Phys. B 2016, 25 (1), 016101 10.1088/1674-1056/25/1/016101.

[ref26] Dong D.; Sälzer F.; Roling B.; Bedrov D. How Efficient Is Li^+^ Ion Transport in Solvate Ionic Liquids under Anion-Blocking Conditions in a Battery?. Phys. Chem. Chem. Phys. 2018, 20 (46), 29174–29183. 10.1039/C8CP06214E. 30426990

[ref27] Nilsson V.; Bernin D.; Brandell D.; Edström K.; Johansson P. Interactions and Transport in Highly Concentrated LiTFSI-based Electrolytes. ChemPhysChem 2020, 21, 1166 10.1002/cphc.202000153. 32311226

[ref28] Newman J.; Thomas-Alyea K. E. Electrochemical Systems, 3rd illustr. ed.; Electrochemical Society series; John Wiley & Sons, 2004.

[ref29] Bizeray A. M.; Howey D. A.; Monroe C. W. Resolving a Discrepancy in Diffusion Potentials, with a Case Study for Li-Ion Batteries. J. Electrochem. Soc. 2016, 163 (8), E223–E229. 10.1149/2.0451608jes.

[ref30] Richardson G. W.; Foster J. M.; Ranom R.; Please C. P.; Ramos A. M. Charge Transport Modelling of Lithium Ion Batteries. arXiv [physics.chem-ph] 2020, 2002.00806.

[ref31] Darken L. S. Diffusion, Mobility and Their Interrelation through Free Energy in Binary Metallic Systems. Trans. Am. Inst. Min. Metall. Eng. 1948, 175 (3), 184–201.

[ref32] Goyal P.; Monroe C. W. Thermodynamic Factors for Locally Non-Neutral, Concentrated Electrolytic Fluids. Electrochim. Acta 2021, 371, 137638 10.1016/j.electacta.2020.137638.

[ref33] Newman J.; Chapman T. W. Restricted Diffusion in Binary Solutions. AIChE J. 1973, 19 (2), 343–348. 10.1002/aic.690190220.

[ref34] Monroe C. W.; Delacourt C. Continuum Transport Laws for Locally Non-Neutral Concentrated Electrolytes. Electrochim. Acta 2013, 114, 649–657. 10.1016/j.electacta.2013.10.006.

[ref35] Liu J.; Monroe C. W. Solute-Volume Effects in Electrolyte Transport. Electrochim. Acta 2014, 135, 447–460. 10.1016/j.electacta.2014.05.009.

[ref36] Wang A.; Hou T.; Karanjavala M.; Monroe C. Shifting-Reference Concentration Cells to Refine Composition-Dependent Transport Characterization of Binary Lithium-Ion Electrolytes. Electrochim. Acta 2020, 358, 136688 10.1016/j.electacta.2020.136688.

[ref37] Hou T.; Monroe C. W. Composition-Dependent Thermodynamic and Mass-Transport Characterization of Lithium Hexafluorophosphate in Propylene Carbonate. Electrochim. Acta 2020, 332, 135085 10.1016/j.electacta.2019.135085.

[ref38] Lundgren H.; Scheers J.; Behm M.; Lindbergh G. Characterization of the Mass-Transport Phenomena in a Superconcentrated LiTFSI:Acetonitrile Electrolyte. J. Electrochem. Soc. 2015, 162 (7), A1334–A1340. 10.1149/2.0961507jes.

[ref39] Lundgren H.; Behm M.; Lindbergh G. Electrochemical Characterization and Temperature Dependency of Mass-Transport Properties of LiPF_6_ in EC:DEC. J. Electrochem. Soc. 2015, 162 (3), A413–A420. 10.1149/2.0641503jes.

[ref40] Farkhondeh M.; Pritzker M.; Fowler M.; Delacourt C. Transport Property Measurement of Binary Electrolytes Using a Four-Electrode Electrochemical Cell. Electrochem. Commun. 2016, 67, 11–15. 10.1016/j.elecom.2016.02.025.

[ref41] Nyman A.; Behm M.; Lindbergh G. Electrochemical Characterisation and Modelling of the Mass Transport Phenomena in LiPF_6_-EC-EMC Electrolyte. Electrochim. Acta 2008, 53 (22), 6356–6365. 10.1016/j.electacta.2008.04.023.

[ref42] Landesfeind J.; Gasteiger H. A. Temperature and Concentration Dependence of the Ionic Transport Properties of Lithium-Ion Battery Electrolytes. J. Electrochem. Soc. 2019, 166 (14), A3079–A3097. 10.1149/2.0571912jes.

[ref43] Bergstrom H. K.; Fong K. D.; Mccloskey B. D. Interfacial Effects on Transport Coefficient Measurements in Li-Ion Battery Electrolytes. J. Electrochem. Soc. 2021, 168, 060543 10.1149/1945-7111/ac0994.

[ref44] Liu J.; Monroe C. W. Solute-Volume Effects in Electrolyte Transport. Electrochim. Acta 2014, 135, 447–460. 10.1016/j.electacta.2014.05.009.

[ref45] Novev J. K.; Compton R. G. Natural Convection Effects in Electrochemical Systems. Curr. Opin. Electrochem. 2018, 7 (1), 118–129. 10.1016/j.coelec.2017.09.010.

[ref46] Selman J. R.; Newman J. Free-Convection Mass Transfer with a Supporting Electrolyte. J. Electrochem. Soc. 1971, 118 (7), 1070–1078. 10.1149/1.2408249.

[ref47] Klett M.; Giesecke M.; Nyman A.; Hallberg F.; Lindström R. W.; Lindbergh G.; Furó I. Quantifying Mass Transport during Polarization in a Li Ion Battery Electrolyte by in Situ ^7^Li NMR Imaging. J. Am. Chem. Soc. 2012, 134 (36), 14654–14657. 10.1021/ja305461j. 22900791

[ref48] Fawdon J.; Ihli J.; Mantia F. L.; Pasta M. Characterising Lithium-Ion Electrolytes via Operando Raman Microspectroscopy. Nat. Commun. 2021, 12 (1), 4053 10.1038/s41467-021-24297-0. 34193848 PMC8245635

[ref49] Takamatsu D.; Yoneyama A.; Asari Y.; Hirano T. Quantitative Visualization of Salt Concentration Distributions in Lithium-Ion Battery Electrolytes during Battery Operation Using X-Ray Phase Imaging. J. Am. Chem. Soc. 2018, 140 (5), 1608–1611. 10.1021/jacs.7b13357. 29334738

[ref50] Bazak J. D.; Allen J. P.; Krachkovskiy S. A.; Goward G. R. Mapping of Lithium-Ion Battery Electrolyte Transport Properties and Limiting Currents with In Situ MRI. J. Electrochem. Soc. 2020, 167 (14), 140518 10.1149/1945-7111/abc0c9.

[ref51] Sethurajan A.; Krachkovskiy S.; Goward G.; Protas B. Bayesian Uncertainty Quantification in Inverse Modeling of Electrochemical Systems. J. Comput. Chem. 2019, 40 (5), 740–752. 10.1002/jcc.25759.

[ref52] Krachkovskiy S. A.; Bazak J. D.; Werhun P.; Balcom B. J.; Halalay I. C.; Goward G. R. Visualization of Steady-State Ionic Concentration Profiles Formed in Electrolytes during Li-Ion Battery Operation and Determination of Mass-Transport Properties by in Situ Magnetic Resonance Imaging. J. Am. Chem. Soc. 2016, 138 (25), 7992–7999. 10.1021/jacs.6b04226. 27250238

[ref53] Bazak J. D.; Krachkovskiy S. A.; Goward G. R. Multi-Temperature in Situ Magnetic Resonance Imaging of Polarization and Salt Precipitation in Lithium-Ion Battery Electrolytes. J. Phys. Chem. C 2017, 121 (38), 20704–20713. 10.1021/acs.jpcc.7b07218.

[ref54] Sethurajan A. K.; Krachkovskiy S. A.; Halalay I. C.; Goward G. R.; Protas B. Accurate Characterization of Ion Transport Properties in Binary Symmetric Electrolytes Using In Situ NMR Imaging and Inverse Modeling. J. Phys. Chem. B 2015, 119 (37), 12238–12248. 10.1021/acs.jpcb.5b04300. 26247105

[ref55] Richardson G.; Foster J. M.; Sethurajan A. K.; Krachkovskiy S. A.; Halalay I. C.; Goward G. R.; Protas B. The Effect of Ionic Aggregates on the Transport of Charged Species in Lithium Electrolyte Solutions. J. Electrochem. Soc. 2018, 165 (9), H561–H567. 10.1149/2.0981809jes.

[ref56] Sethurajan A. K.; Foster J. M.; Richardson G.; Krachkovskiy S. A.; Bazak J. D.; Goward G. R.; Protas B. Incorporating Dendrite Growth into Continuum Models of Electrolytes: Insights from NMR Measurements and Inverse Modeling. J. Electrochem. Soc. 2019, 166 (8), A1591–A1602. 10.1149/2.0921908jes.

[ref57] Ma Y.; Doyle M.; Fuller T. F.; Doeff M. M.; De Jonghe L. C.; Newman J. The Measurement of a Complete Set of Transport Properties for a Concentrated Solid Polymer Electrolyte Solution. J. Electrochem. Soc. 1995, 142 (6), 1859–1868. 10.1149/1.2044206.

[ref58] Kondo K.; Sano M.; Hiwara A.; Omi T.; Fujita M.; Kuwae A.; Iida M.; Mogi K.; Yokoyama H. Conductivity and Solvation of Li^+^ Ions of LiPF_6_ in Propylene Carbonate Solutions. J. Phys. Chem. B 2000, 104 (20), 5040–5044. 10.1021/jp000142f.

[ref59] Hwang S.; Kim D. H.; Shin J. H.; Jang J. E.; Ahn K. H.; Lee C.; Lee H. Ionic Conduction and Solution Structure in LiPF_6_ and LiBF_4_ Propylene Carbonate Electrolytes. J. Phys. Chem. C 2018, 122 (34), 19438–19446. 10.1021/acs.jpcc.8b06035.

[ref60] Han S. D.; Yun S. H.; Borodin O.; Seo D. M.; Sommer R. D.; Young V. G.; Henderson W. A. Solvate Structures and Computational/Spectroscopic Characterization of LiPF_6_ Electrolytes. J. Phys. Chem. C 2015, 119 (16), 8492–8500. 10.1021/acs.jpcc.5b00826.

[ref61] Heckmann A.; Thienenkamp J.; Beltrop K.; Winter M.; Brunklaus G.; Placke T. Towards High-Performance Dual-Graphite Batteries Using Highly Concentrated Organic Electrolytes. Electrochim. Acta 2018, 260, 514–525. 10.1016/j.electacta.2017.12.099.

[ref62] Cresce A. V.; Russell S. M.; Borodin O.; Allen J. A.; Schroeder M. A.; Dai M.; Peng J.; Gobet M. P.; Greenbaum S. G.; Rogers R. E.; Xu K. Solvation Behavior of Carbonate-Based Electrolytes in Sodium Ion Batteries. Phys. Chem. Chem. Phys. 2017, 19 (1), 574–586. 10.1039/C6CP07215A. 27918030

[ref63] Katon J. E.; Cohen M. D. The Vibrational Spectra and Structure of Dimethyl Carbonate and Its Conformational Behavior. Can. J. Chem. 1975, 53 (9), 1378–1386. 10.1139/v75-191.

[ref64] Haneke L.; Frerichs J. E.; Heckmann A.; Lerner M. M.; Akbay T.; Ishihara T.; Hansen M. R.; Winter M.; Placke T. Mechanistic Elucidation of Anion Intercalation into Graphite from Binary-Mixed Highly Concentrated Electrolytes via Complementary ^19^F MAS NMR and XRD Studies. J. Electrochem. Soc. 2020, 167 (14), 140526 10.1149/1945-7111/abc437.

[ref65] Seo D. M.; Reininger S.; Kutcher M.; Redmond K.; Euler W. B.; Lucht B. L. Role of Mixed Solvation and Ion Pairing in the Solution Structure of Lithium Ion Battery Electrolytes. J. Phys. Chem. C 2015, 119 (25), 14038–14046. 10.1021/acs.jpcc.5b03694.

[ref66] Self J.; Fong K. D.; Persson K. A. Transport in Superconcentrated LiPF 6 and LiBF 4 /Propylene Carbonate Electrolytes. ACS Energy Letters 2019, 4, 2843 10.1021/acsenergylett.9b02118.

[ref67] Yoon H.; Howlett P. C.; Best A. S.; Forsyth M.; MacFarlane D. R. Fast Charge/Discharge of Li Metal Batteries Using an Ionic Liquid Electrolyte. J. Electrochem. Soc. 2013, 160 (10), A1629–A1637. 10.1149/2.022310jes.

[ref68] Bruce P. G.; Evans J.; Vincent C. A. Conductivity and Transference Number Measurements on Polymer Electrolytes. Solid State Ionics 1988, 28–30, 918–922. 10.1016/0167-2738(88)90304-9.

[ref69] Balsara N. P.; Newman J. Relationship between Steady-State Current in Symmetric Cells and Transference Number of Electrolytes Comprising Univalent and Multivalent Ions. J. Electrochem. Soc. 2015, 162 (14), A2720–A2722. 10.1149/2.0651514jes.

[ref70] Robinson R. A.; Stokes R. H. Electrolyte Solutions: Second Revised ed., 2nd ed.; Dover Publications, Inc., 2012.

[ref71] Klamor S.; Zick K.; Oerther T.; Schappacher F. M.; Winter M.; Brunklaus G. 7Li in Situ 1D NMR Imaging of a Lithium Ion Battery. Phys. Chem. Chem. Phys. 2015, 17 (6), 4458–4465. 10.1039/C4CP05021E. 25578436

[ref72] Wang A. A. EMC MRI model. https://github.com/ndrewwang/EMC-MRI-model. 10.5281/zenodo.4767883.

[ref73] Reniers J. M.; Mulder G.; Howey D. A. Review and Performance Comparison of Mechanical-Chemical Degradation Models for Lithium-Ion Batteries. J. Electrochem. Soc. 2019, 166 (14), A3189–A3200. 10.1149/2.0281914jes.

[ref74] Liu J.; Monroe C. W. On the Characterization of Battery Electrolytes with Polarization Cells. Electrochim. Acta 2015, 167, 357–363. 10.1016/j.electacta.2015.03.104.

[ref75] Stewart S. G.; Newman J. The Use of UV/Vis Absorption to Measure Diffusion Coefficients in LiPF_6_ Electrolytic Solutions. J. Electrochem. Soc. 2008, 155 (1), F13 10.1149/1.2801378.

[ref76] Thompson S. D. Differential Diffusion Coefficients of Sodium Polysulfide Melts. J. Electrochem. Soc. 2006, 136 (11), 3362 10.1149/1.2096451.

[ref77] Ehrl A.; Landesfeind J.; Wall W. A.; Gasteiger H. A. Determination of Transport Parameters in Liquid Binary Lithium Ion Battery Electrolytes. J. Electrochem. Soc. 2017, 164 (4), A826–A836. 10.1149/2.1131704jes.

[ref78] Wood K. N.; Kazyak E.; Chadwick A. F.; Chen K. H.; Zhang J. G.; Thornton K.; Dasgupta N. P. Dendrites and Pits: Untangling the Complex Behavior of Lithium Metal Anodes through Operando Video Microscopy. ACS Cent. Sci. 2016, 2 (11), 790–801. 10.1021/acscentsci.6b00260. 27924307 PMC5126712

[ref79] Chen K. H.; Wood K. N.; Kazyak E.; Lepage W. S.; Davis A. L.; Sanchez A. J.; Dasgupta N. P. Dead Lithium: Mass Transport Effects on Voltage, Capacity, and Failure of Lithium Metal Anodes. J. Mater. Chem. A 2017, 5 (23), 11671–11681. 10.1039/C7TA00371D.

[ref80] Mistry A.; Fear C.; Carter R.; Love C. T.; Mukherjee P. P. Electrolyte Confinement Alters Lithium Electrodeposition. ACS Energy Lett. 2019, 4, 156–162. 10.1021/acsenergylett.8b02003.

[ref81] Ilott A. J.; Mohammadi M.; Chang H. J.; Grey C. P.; Jerschow A. Real-Time 3D Imaging of Microstructure Growth in Battery Cells Using Indirect MRI. Proc. Natl. Acad. Sci. U. S. A. 2016, 113 (39), 10779–10784. 10.1073/pnas.1607903113. 27621444 PMC5047163

[ref82] Ilott A. J.; Chandrashekar S.; Klöckner A.; Chang H. J.; Trease N. M.; Grey C. P.; Greengard L.; Jerschow A. Visualizing Skin Effects in Conductors with MRI: ^7^Li MRI Experiments and Calculations. J. Magn. Reson. 2014, 245, 143–149. 10.1016/j.jmr.2014.06.013. 25036296

[ref83] Xu S.; Harel E.; Michalak D. J.; Crawford C. W.; Budker D.; Pines A. Flow in Porous Metallic Materials: A Magnetic Resonance Imaging Study. J. Magn. Reson. Imaging 2008, 28 (5), 1299–1302. 10.1002/jmri.21532. 18972341

[ref84] Bazak J. D.; Allen J. P.; Krachkovskiy S. A.; Goward G. R. Mapping of Lithium-Ion Battery Electrolyte Transport Properties and Limiting Currents with In Situ MRI. J. Electrochem. Soc. 2020, 167 (14), 140518 10.1149/1945-7111/abc0c9.

[ref85] Stewart S.; Newman J. Measuring the Salt Activity Coefficient in Lithium-Battery Electrolytes. J. Electrochem. Soc. 2008, 155 (6), A458 10.1149/1.2904526.

[ref86] Tjaden B.; Cooper S. J.; Brett D. J.; Kramer D.; Shearing P. R. On the Origin and Application of the Bruggeman Correlation for Analysing Transport Phenomena in Electrochemical Systems. Curr. Opin. Chem. Eng. 2016, 12, 44–51. 10.1016/j.coche.2016.02.006.

[ref87] Richardson G.; Korotkin I.; Ranom R.; Castle M.; Foster J. M. Generalised Single Particle Models for High-Rate Operation of Graded Lithium-Ion Electrodes: Systematic Derivation and Validation. Electrochim. Acta 2020, 339, 135862 10.1016/j.electacta.2020.135862.

[ref88] Monroe C.; Newman J. Dendrite Growth in Lithium/Polymer Systems. J. Electrochem. Soc. 2003, 150 (10), A1377 10.1149/1.1606686.

[ref89] Kushima A.; So K. P.; Su C.; Bai P.; Kuriyama N.; Maebashi T.; Fujiwara Y.; Bazant M. Z.; Li J. Liquid Cell Transmission Electron Microscopy Observation of Lithium Metal Growth and Dissolution: Root Growth, Dead Lithium and Lithium Flotsams. Nano Energy 2017, 32, 271–279. 10.1016/j.nanoen.2016.12.001.

[ref90] Liu Y.; Xu X.; Sadd M.; Kapitanova O. O.; Krivchenko V. A.; Ban J.; Wang J.; Jiao X.; Song Z.; Song J.; Xiong S.; Matic A. Insight into the Critical Role of Exchange Current Density on Electrodeposition Behavior of Lithium Metal. Adv. Sci. 2021, 8 (5), 2003301 10.1002/advs.202003301. PMC7927631 33717853

[ref91] As discussed earlier by the authors, the superconcentrated LiPF_6_:EMC electrolyte studied here should be amenable to description by eqs 1 and 2, so long as all local association/dissociation reactions reach equilibrium on a time scale much faster than the characteristic time scale for diffusion.^36,55^

