# Supplementary material for: Potentiometric MRI of a Superconcentrated Lithium Electrolyte: Testing the Irreversible Thermodynamics Approach
Source: ACS Energy Lett. 2021 Aug 15;6(9):3086–95. doi: 10.1021/acsenergylett.1c01213 (PMC8438662; doi:10.1021/acsenergylett.1c01213)
Supplement: Supplementary file 1 — nz1c01213_si_001.pdf [file nz1c01213_si_001.pdf]

Supporting Information:

Potentiometric MRI of a Superconcentrated Lithium Electrolyte: Testing the Irreversible Thermodynamics Approach

Andrew A. Wang[1,2], *Anna B. Gunnarsdóttir*[3], *Jack Fawdon*[4], *Mauro Pasta*[2,4], *Clare P. Grey*[2,3], *Charles W. Monroe*[1,2]*

[1] Department of Engineering Science, University of Oxford, Oxford OX1 3PJ, U.K.
[2] The Faraday Institution, Harwell Campus, Didcot OX11 0RA, U.K.
[3] Department of Chemistry, University of Cambridge, Cambridge CB2 1EW, U.K.
[4] Department of Materials Science, University of Oxford, Oxford OX1 3PH, U.K.

* Correspondence: charles.monroe@eng.ox.ac.uk

Table of Contents

S1. Experimental Procedures 2

A. Preparation of $LiPF_6$:EMC solutions 2

B. Ex-situ transport and thermodynamic property characterization 2

C. Raman spectroscopy 5

D. Potentiometric MRI 5

S2. Supporting Discussion 7

A. Transport model formulation with solute volume effects 7

B. Partial molar volume, composition bases, thermodynamic factor 8

C. Transport and thermodynamic property correlations 9

D. Onsager–Stefan–Maxwell diffusion coefficients 10

E. Raman spectra 12

F. In-situ MRI experiment 14

G. Electrochemical response and surface kinetics analysis 19

H. Dynamic Viscosities 22

I. Other Raw data 23

J. References 25

S1. Experimental Procedures

A. Preparation of $LiPF_6$:EMC solutions

Electrolyte solutions were formulated on a mass fraction basis in a glovebox (Inert Technologies) under argon gas (H_2O < 1 ppm, O_2 < 1 ppm). $LiPF_6$ salt (99.99%, battery grade, Sigma Aldrich) was dried in a heated vacuum antechamber for 24 hours at 60 ºC, and EMC (99.9%, anhydrous) was dried under 3 Å molecular sieves for a week. Solutions were mixed with magnetic stir bars in polypropylene bottles for over 48 hours to ensure full dissolution. Moisture content was determined by Karl Fischer titration to be below 10 ppm.

B. Ex-situ transport and thermodynamic property characterization

Parameterization of transport and thermodynamic properties followed experimental procedures that have been detailed in previous publications.[1,2] Below we summarize the key methods: Solution densities were measured with a temperature-controlled oscillating densitometer (DMA4100, Anton Paar) within the glovebox. Ionic conductivity was measured with an AC probe (Orion A212, Thermo Scientific) in a sealed, liquid-tight cell placed in a temperature-controlled water bath at 25 ºC.

Li-Li coin cells with 2 mm thick annular PEEK spacers were assembled and restricted-diffusion experiments were performed in a 25 °C thermal chamber.[3] The annular gap was packed with a glass fibre separator. Effective Fickian diffusivities were extracted from the voltage decay at open circuit after application of a 100 mV hold for 12 hours, according to

$$\lim_{t \to \inf} \frac{d \ln(\mathrm{OCP})}{dt} = -\frac{\pi^2 D_{\mathrm{eff}}}{L^2 \varepsilon}, \tag{S1}$$

where ε is the separator porosity in the coin cell. Figure S1 shows a representative voltage trace and fitting. The chosen annular spacer thickness and potentiostatic hold ensure a less noisy, longer relaxation period, as discussed earlier by Wang et al. [1]

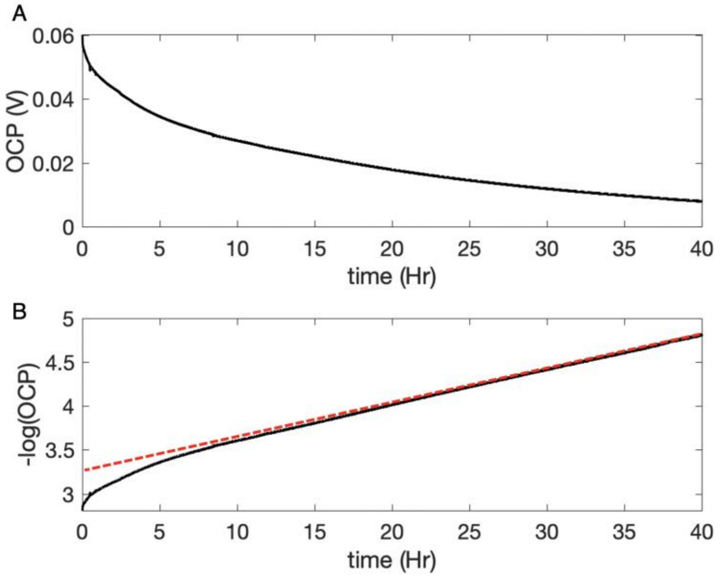

Figure S1. (A) Representative open circuit voltametric response during restricted diffusion experiment, and (B) shows the semi-log response with the fitted linear decay region (red - - -).

Custom Hittorf cells with three chambers separated by two valves were used to determine transference numbers, by recording the relative changes in solution density of the anodic and cathodic chambers relative to the central chamber. After passing 14.4 C of charge (Q), both valves were closed and the solution density within each chamber was measured after combing to equilibrium. Hittorf measurements were completed within the glovebox, thermostatted at 25 ºC. The transference number was calculated from the change in moles (Δn) of lithium moved relative to the initial bulk concentration (c_∞) during the experiment:

$$t_+^0 = 1 - \frac{\Delta n F}{Q(1 - c_\infty \bar{V}_\mathrm{e})}. \qquad \text{(S2)}$$

A derivation and error analysis for equation S2 was provided by Hou and Monroe.[2] Below we illustrate an exemplary schematic of the concentration profiles during the Hittorf experiment.

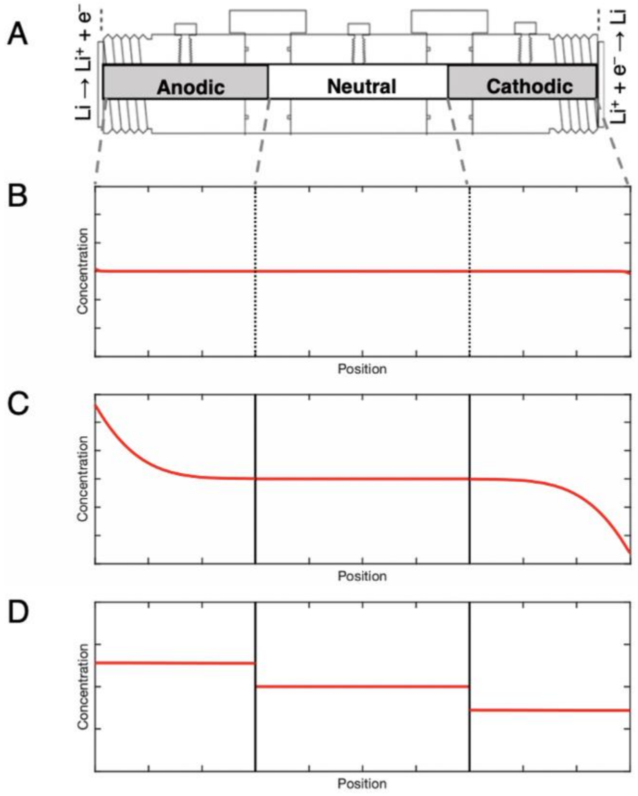

Figure S2. (A) Schematic of the valved Hittorf cell with anodic, neutral, and cathodic electrolyte chambers. (B) represents the initial concentration profile, followed by the gradient during polarization depicted in (C) and finally the resting concentrations after current and valves are shut and each chamber is allowed to settle after mixing (D).

Finally, fritted concentration cells with an "H" geometry were used to determine liquid-junction potentials. A matrix of test concentrations was probed using a shifting reference concentration, as described in detail by Wang et al.[1]

C. Raman spectroscopy

A Renishaw inVia Reflex laser confocal Raman microscope equipped with a near-IR 785 nm laser and a 5x magnification objective (Leica, 0.12 NA, 14 WD) was used to gather Raman spectra for intermolecular structural characterization. Specifically, full scans were performed from 1600 cm^{-1} to 700 cm^{-1}, with a 5% laser power and 10 second exposure time to identify bands of note. Following acquisition, the data was processed using Renishaw WiRE 5.5 software and the background signal was removed. Spectra were then imported into Origin Pro software for further analysis and peak fitting (see supporting discussion S2.E).

D. Potentiometric MRI

The custom Swagelok-type PEEK cell used in the MRI experiments consisted of a cylindrical cell with 4 mm inner diameter and column length of 8 mm, PEEK screw caps, machined Cu plungers as current collectors and Viton o-rings for sealing (see schematic, figure S7). The cell was assembled with two pieces of Li metal (LTS research, 99.95%) cut into cylindrical discs with a puncher, 4mm in diameter, adhered to the Cu current collector. The electrolyte loading was 130 μL, more than the cell volume of 100 μL, to avoid formation of gas bubbles. The excess electrolyte is displaced by the lithium electrode as the cell is sealed (see Fig. S8). The PEEK cell was connected to shielded coaxial cables going through the open MicWB40 probe. Outside the probe, the cables were connected to a low-pass filter with 5 MHz cut-off to minimize noise pickup by the rf-coil. Additional shielded coaxial cables then connected to the potentiostat.

Electrochemical measurements were performed using a Biologic SP-150 potentiostat with EC-Laboratory software. The MRI cell was preconditioned with a cycling protocol consisting of +4

μA for one minute, and –4 μA for one minute, with 5 repeats. This was done to encourage electrochemical SEI formation before the potentiometric MRI. After a rest period, the final open-circuit potential recorded for 30 min (13 mV) was used to offset the recorded voltage behavior during polarization and relaxation to ensure compatibility with COMSOL assumptions.[4]

60 μA (0.48 mA/cm^2) was then applied to the MRI cell for 2 hours, followed by a measurement of the open-circuit relaxation for 8 hours. The constant current was orientated so that Li plating occurred on the top electrode, to suppress free convection due to induced density gradients.[5]

The ^{19}F MRI experiments were performed on a Bruker Avance III 300 spectrometer operating at 7.05 T (^{19}F frequency = 282.4 MHz). The spectra were acquired with a MicWB40 probe and a Micro2.5 triple axis gradient system at 298 K, using a water-cooler unit, and 10 mm exchangeable ^{1}H-^{19}F/^{7}Li coil. The cell was aligned such the Li metal electrodes were perpendicular to the external magnetic B_0-field (and to the z-axis of the gradient).

One-dimensional image profiles were acquired axially (in the z-direction) to get concentration profiles. The image profiles were acquired using the 1D spin-echo sequence (diffprof) reported by Klamor et al.[6] 64 scans were acquired with a recycle delay of 4 s, which resulted in acquisition time of 4.25 min for one image profile. The 90° pulse length was set to 18 μs and a gradient strength of 22.5 G cm^{-1}. The field of view (FOV) was 20 mm, resulting in a nominal resolution of 19.5 μm per point. The duration between the 90° pulse and the composite 180° pulse was 1.1 ms, yielding an echo time of 2.2 ms (T_2 = 300 ms). The gradient stabilization time was set to 100 μs and the dephasing time to 800 μs. The cells were aligned in the centre of the

coil to ensure uniform excitation. An RF sensitivity profile was measured on an NMR tube filled with the electrolyte, shown in Fig. S8.

S2. Supporting Discussion

A. Transport model formulation with solute volume effects

The polarization-cell model implemented in COMSOL Multiphysics software follows that outlined by Hou and Monroe.[2]

Constitutive laws and balances:

$$\vec{N}_+ = -D\vec{\nabla}c + \frac{t_+^0}{F}\vec{i} + c\vec{v}^\square \tag{S3}$$

$$\vec{v}^\square = \bar{V}_\mathrm{e}\left[t_-^0\vec{N}_+ + t_+^0\vec{N}_-\right] + \bar{V}_0\vec{N}_0 \tag{S4}$$

$$-\vec{\nabla}\cdot\vec{v}^\square = \frac{\bar{V}_\mathrm{e}}{F}\vec{i}\cdot\vec{\nabla}t_+^0 + \frac{D}{1-\bar{V}_\mathrm{e}c}\vec{\nabla}c\cdot\vec{\nabla}\bar{V}_\mathrm{e} \tag{S5}$$

$$\vec{\nabla}\cdot\vec{i} = 0 \tag{S6}$$

$$\frac{\partial c}{\partial t} = -\vec{\nabla}\cdot\vec{N}_+ \tag{S7}$$

$$\vec{\nabla}\Phi = -\frac{\vec{i}}{\kappa} + \frac{2RT\chi(1-t_+^0)}{F\left[1+(2\bar{V}_0-\bar{V}_\mathrm{e})c\right]}\vec{\nabla}\ln c \tag{S8}$$

Boundary conditions:

$$\vec{i} = \frac{I(t)}{A} \tag{S9}$$

$$\vec{N}_+\big|_{x=0} = \frac{I(t)}{FA} \tag{S10}$$

$$\left.\frac{\vec{v}^\square}{\overline{V}_\mathrm{e}\left(1-t_+^0\right)}\right|_{x=0} = \frac{I(t)}{FA} \tag{S11}$$

Initial condition:

$$c(0,x) = c^\mathrm{eq} \tag{S12}$$

B. Partial molar volume, composition bases, thermodynamic factor

Partial molar volumes for solvent $\bar{V}_0$ and solute $\bar{V}_e$ plotted on figure 1E are calculated by examining how solution density changes as a function of salt concentration. The following expressions extract partial molar volumes from the density/molarity correlation:[7]

$$\overline{V}_0 = \frac{M_0}{\rho - c\frac{d\rho}{dc}} \tag{S13}$$

$$\overline{V}_\mathrm{e} = \frac{M_\mathrm{e} - \frac{d\rho}{dc}}{\rho - c\frac{d\rho}{dc}} . \tag{S14}$$

Here M_0 is the molar mass of EMC (104.105 g/mol) and M_e is the molar mass of $LiPF_6$ (151.905 g/mol). Electrolytes in parameterization experiments use a variety of composition bases including molarity c, mass fraction ω, and cation particle fraction y. They may be converted based on the following expressions for binary electrolytic solutions:

$$c = \frac{\omega\rho}{M_\mathrm{e}} \tag{S15}$$

$$y = \frac{M_0 c}{\rho + (2M_0 - M_\mathrm{e})\, c} = \frac{M_0 \omega}{M_\mathrm{e} + (2M_0 - M_\mathrm{e})\, \omega} . \tag{S16}$$

The thermodynamic factor χ expresses how the salt's activity in the liquid varies with its concentration. In terms of salt activity coefficients, χ can be written as

$$\chi = 1 + \left(\frac{\partial \ln \lambda_{+-}}{\partial \ln y}\right)_{T,p} = \frac{1}{y_0}\left[1 + \left(\frac{\partial \ln \gamma_{+-}}{\partial \ln m}\right)_{T,p}\right] = \frac{\bar{V}_0}{y_0}\left[1 + \left(\frac{\partial \ln f_{+-}}{\partial \ln c}\right)_{T,p}\right], \tag{S17}$$

in which m is salt molality and c is salt molarity; $\lambda_{+-}, \gamma_{+-},$ and f_{+-} respectively represent mean molar salt activity coefficients over particle-fraction, molal, and molar bases.

C. Transport and thermodynamic property correlations

Below is a summary of property correlations used in the transport model, arrived at by following the same fitting procedure described by Wang et al.[1] and the experimental data presented in Fig. 2. The following expressions are valid for $LiPF_6$:EMC up to 3.8 M at 25 ºC.

Table S1. Correlations

Property	Correlation	Units
$\rho(c\ [M])$	$1007.1 + 114.2c - 8.121c^{\frac{3}{2}} - 4.013 \times 10^{-5}c^{10}$	gL^{-1}
$\kappa(y)$	$\left(48.93y^{\frac{3}{2}} - 284.8y^{\frac{5}{2}} + 817.7y^{4}\right)^{2}$	Sm^{-1}
$D(y)$	$(4.998 - 29.96y + 53.78y^{2}) \times 10^{-10}$	m^2s^{-1}
$t_{+}^{0}(y)$	$0.4107 - 1.487y + 2.547y^{2}$	—
$\chi(y)$	$1 - 18.38y^{\frac{1}{2}} + 155.3y - 450.6y^{\frac{3}{2}} + 1506y^{\frac{5}{2}}$	—

Table S2. Coefficient values from Table S1 with 95% confidence intervals

Property	Coefficients			
$\rho(c\ [M])$	114.2 ± 2.3	-8.121 ± 1.527	-4.013×10^{-5} $\pm 0.779 \times 10^{-5}$	
$\kappa(y)$	48.93 ± 0.61	-284.8 ± 5.6	817.7 ± 28.7	
$D(y)$	4.998 ± 0.201	-29.96 ± 3.84	-53.78 ± 14.84	
$t_{+}^{0}(y)$	0.4107 ± 0.0201	-1.487 ± 0.414	2.547 ± 1.717	
$\chi(y)$	-18.38 ± 12	155.3 ± 111.5	-450.6 ± 276.7	1506 ± 422

D. Onsager–Stefan–Maxwell diffusion coefficients

OSM diffusivities for the binary monovalent electrolyte $LiPF_6$:EMC can be mapped from bulk transport properties through

$$\mathscr{D}_{0-} = \frac{\mathscr{D}}{2t_+^0} \tag{S18}$$

$$\mathscr{D}_{0+} = \frac{\mathscr{D}}{2(1-t_+^0)} \tag{S19}$$

$$\frac{1}{\mathscr{D}_{+-}} = \frac{F^2}{RT\Lambda y} - \frac{2(1-2y)t_+^0(1-t_+^0)}{\mathscr{D} y} \tag{S20}$$

where D is the thermodynamic diffusivity, equal to the Fickian diffusivity scaled by the thermodynamic factor, D/χ, and Λ is the equivalent conductance, equal to κ/c.

The extended Stefan–Maxwell equation with electrochemical potential μ_i and species velocity $\vec{v}_i$ for species *i* and *j* is given by:

$$-c_i \vec{\nabla} \mu_i = \sum_{j \neq i} \frac{RT c_i c_j}{c_\mathrm{T} \mathscr{D}_{ij}} (\vec{v}_i - \vec{v}_j) = \sum_{j \neq i} K_{ij} (\vec{v}_i - \vec{v}_j), \tag{S21}$$

where K_{ij} simplifies notation as a diffusional drag coefficient with units of force per volume per velocity, as is plotted below in figure S3A. Ion correlations via the Onsager L matrix may also be computed in terms of drag following the mapping by Fong et al.[8]

The ionic conductivity κ for a lithium electrolyte in the absence of concentration gradients can be identified as

$$\frac{1}{\kappa} = \frac{RT}{F^2 c_\mathrm{T}} \left[\frac{1}{\mathscr{D}_{+-}} + \frac{2c_0 t_+^0 (1 - t_+^0)}{\mathscr{D} c} \right]. \tag{S22}$$

Thus the ionic resistivity can be calculated in terms of fractional contributions from cation-anion interactions and solute-solvent interactions, as plotted in figure S3B and given respectively by

$$\frac{\frac{1}{\mathscr{D}_{+-}}}{\frac{1}{\mathscr{D}_{+-}} + \frac{2c_0 t_+^0 (1-t_+^0)}{\mathscr{D} c}} \quad \text{and} \quad \frac{\frac{2c_0 t_+^0 (1-t_+^0)}{\mathscr{D} c}}{\frac{1}{\mathscr{D}_{+-}} + \frac{2c_0 t_+^0 (1-t_+^0)}{\mathscr{D} c}}. \tag{S23}$$

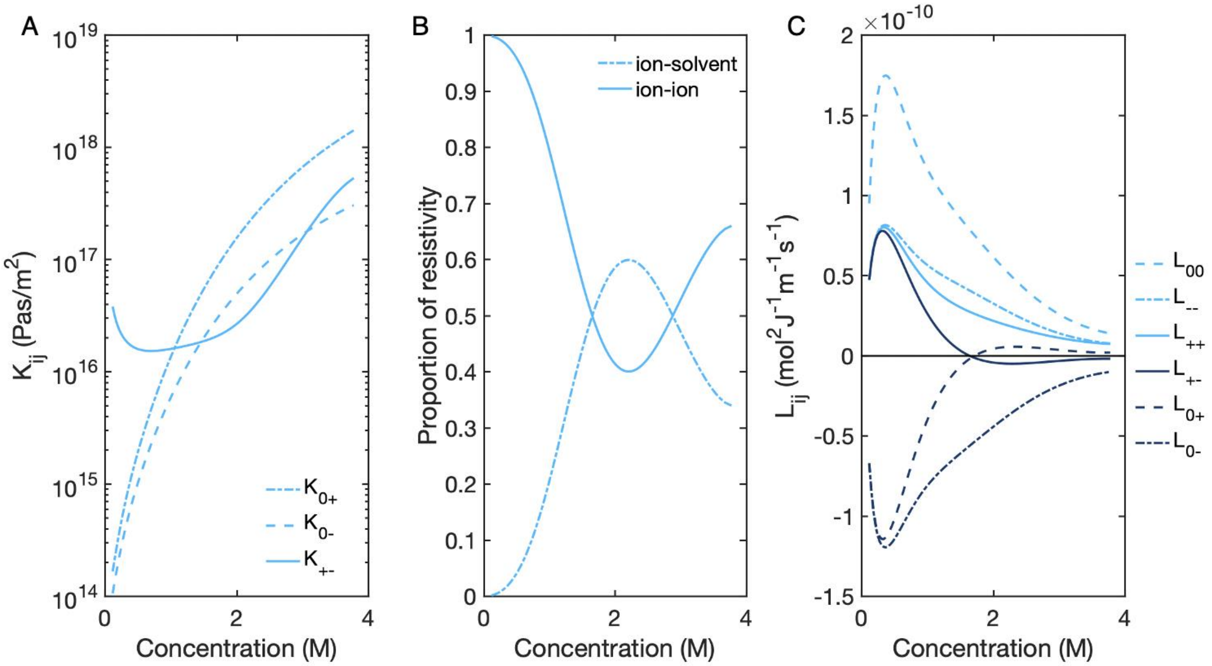

Figure S3. (A) Onsager drag coefficients K_{ij} determined from equation S20, (B) the proportion of ionic resistivity contributed by ion-ion and ion-solvent interactions as calculated from equation S21–22, and (C) Onsager correlation decay rates L_{ij} computed with the inverse of the mapping given by Fong et al.[8]

E. Raman spectra

Full spectra collected for $LiPF_6$:EMC from 0 M to saturation are plotted in figure S4. It is observed that the scattering efficiency of $LiPF_6$ electrolytes in the super-concentrated regime is poor.[9]

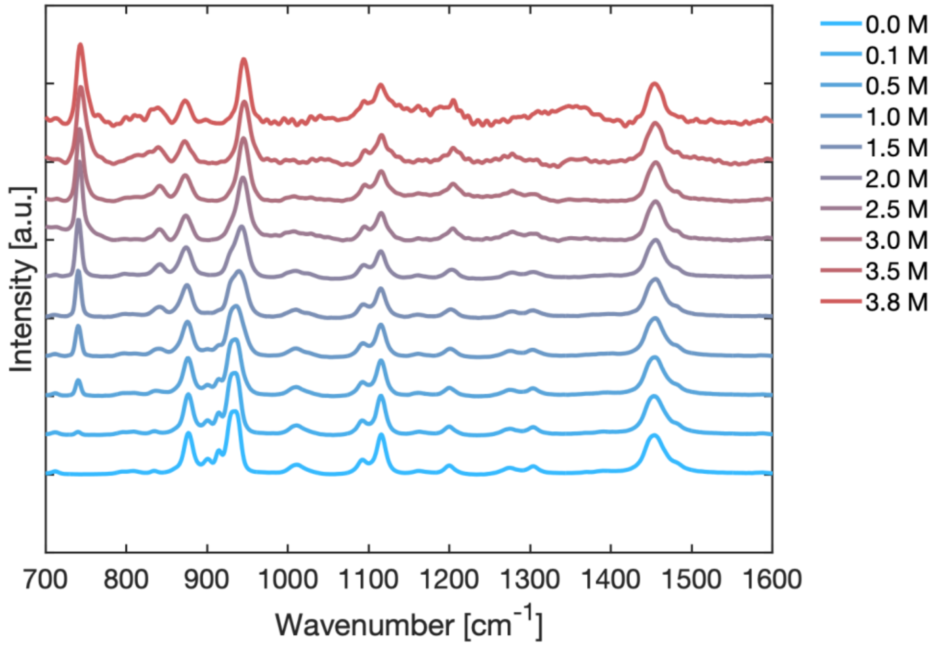

Figure S4. Full Raman spectra for $LiPF_6$:EMC at room temperature. Vertically offset for legibility.

Figure S5 demonstrates the peak area fitting with Voigt functions in Origin Pro. The neat EMC solvent has a characteristically broad peak at 928 and 937 cm^{-1}, which is attributed to the free C–O stretching modes influenced by the asymmetric ethyl and methyl groups in EMC.[10] The coordinated solvent peak is seen to emerge at approx. 946 cm^{-1}, as plotted in figure S5.

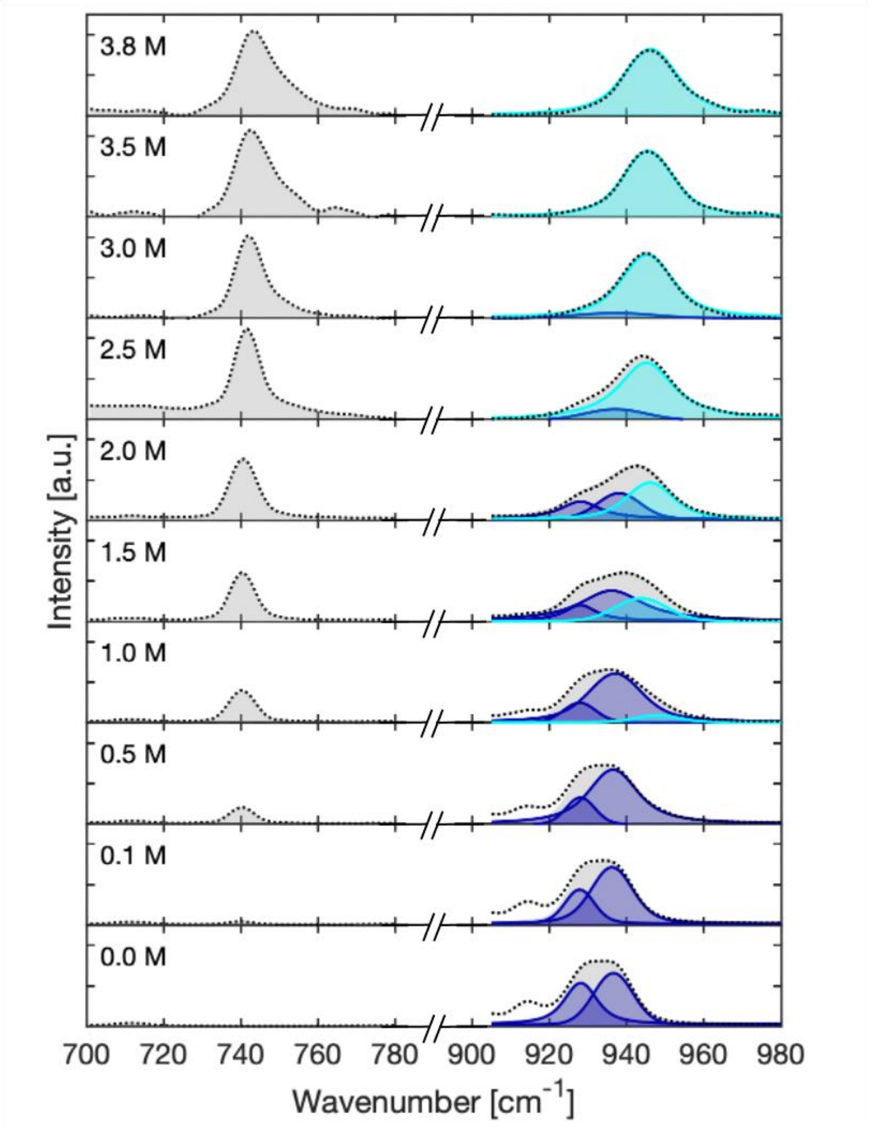

Figure S5. Stacked Raman spectra in the range of 720 – 780 cm^{-1} for PF_6^- and 920 – 980 cm^{-1} for EMC with peak deconvolution fitting for free (navy) and coordinated (cyan) EMC.

Without prescribing specific speciation for PF_6^-, the shifting position of maximum peak height in figure S6 suggests that ion association occurs. It is expected that a quasi-equilibrium exists between solvent-separated ion pairs, contact ion pairs, and higher order aggregates.

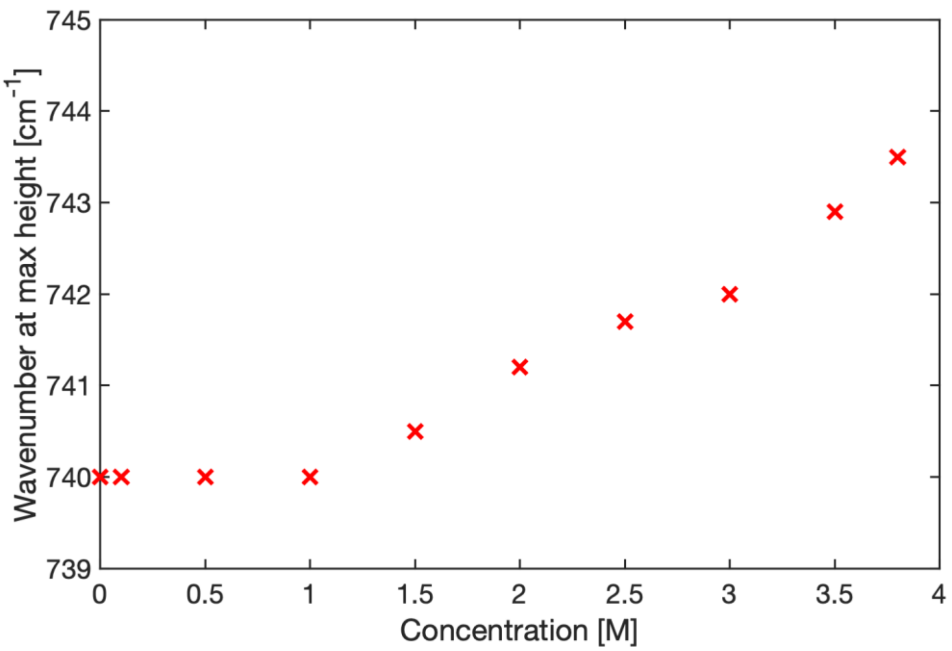

Figure S6. Position of max peak height for PF_6^- with concentration.

F. In-situ MRI experiment

MRI cell schematic

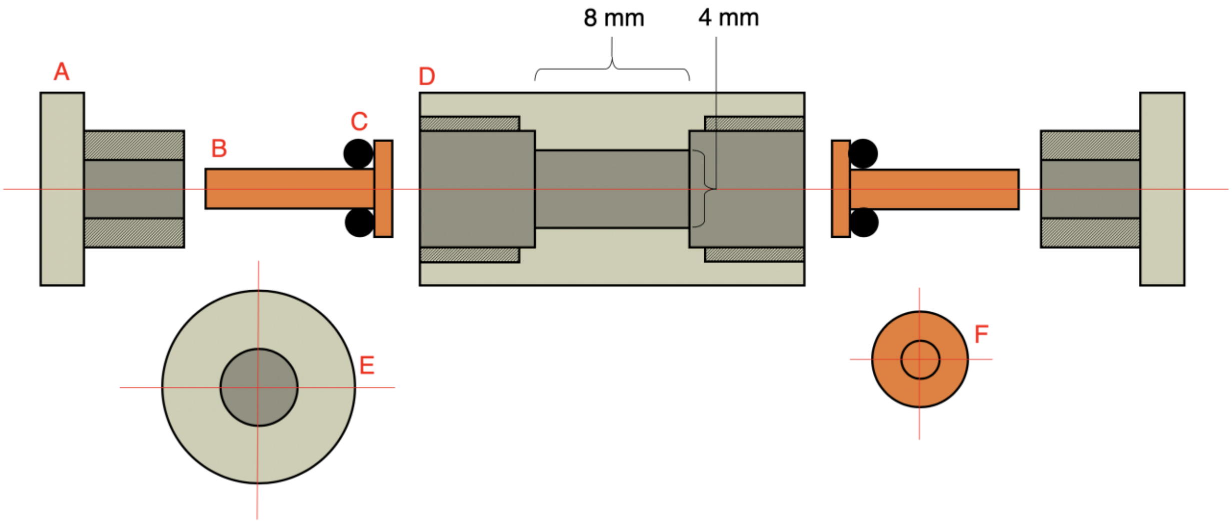

Figure S7. Schematic of the cylindrical MRI cell, with cross-sectional side views of (A) end caps, (B) copper current collectors, (C) Viton O-rings, and (D) cell electrolyte chamber, and top views of (E) the end caps and (F) the copper current collectors.

Calibration and full MRI profile

Inter-electrode spacing was calibrated with a custom PEEK cell with liquid height of 12 mm. This separate cell, sealed without electrodes, removes noise introduced by metal susceptibility effects at the interface.[5] Figure S8 compares the overall RF coil signal of a full NMR tube with that of the calibration cell. Additional intensity peaks beyond the bulk electrolyte are residual electrolyte within the threading of the constructed cell, displaced there during cell sealing.

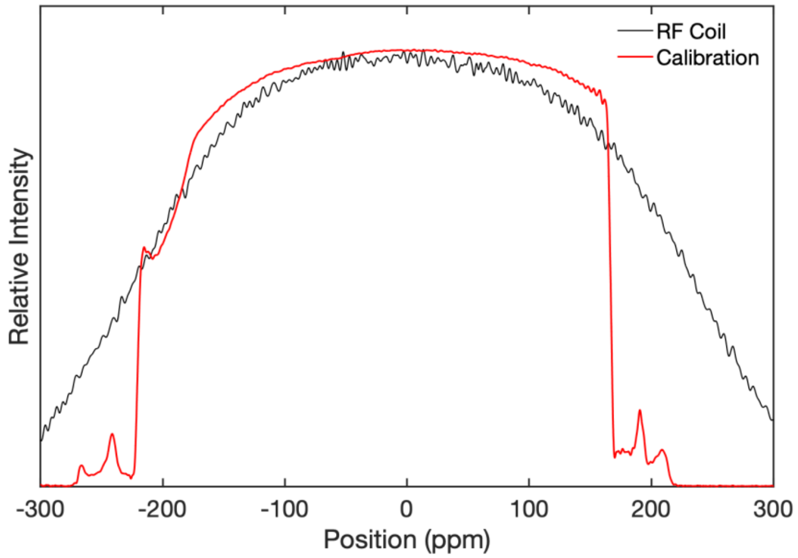

Figure S8 Intensity profile of calibration cell with 12 mm liquid height (red) and overall RF coil signal (black).

Following the attained calibration, the full intensity profile of the MRI cell during the polarization step is also shown below in figure S9.

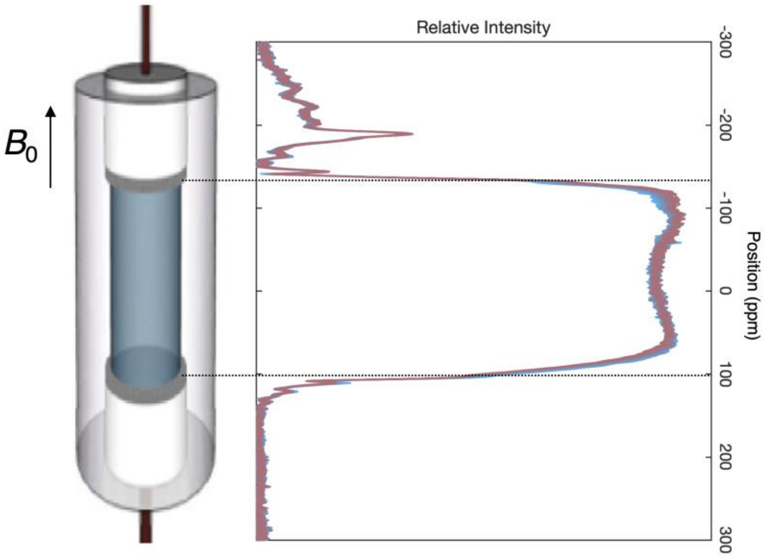

Figure S9. Initial (blue —) and final (red —) full intensity profiles for 3 M polarization step with identified inter-electrode distance (dashed). Magnetic field B_0 orientation is indicated by the arrow with a schematic of the MRI cell.

Estimation of mossy Li growth

For a single polarization experiment, it is expected that any mossy dendritic lithium plated should extend no more than 50 μm into the bulk electrolyte. This estimate is based on the visualization presented in the supporting information by Wood and coworkers for 1 M $LiPF_6$ in EC:DMC.[11] They observed 50 μm of lithium growth was for $1.25\ \mathrm{mAhcm^{-2}}$ of charge passed, at a current density of $5\ \mathrm{mAcm^{-2}}$. In this study, a total of $0.95\ \mathrm{mAhcm^{-2}}$ of Li is plated, at an order-of-magnitude lower current density of $0.48\ \mathrm{mAcm^{-2}}$. Despite this observation, in Fig. 3E, a loss of signal intensity extends roughly 500 μm into the bulk electrolyte from the plated electrode and most likely owes to the combined effects of RF attenuation and magnetic susceptibility. Previous work by Ilott et al. showed that quantifying loss of signal intensity of the electrolyte solvent by MRI led to significant overestimation of Li metal growth.[12]

Low pass filtering of MRI data

A low pass filter was applied to the raw MRI data to dampen higher-frequency noise. For a sampling time of 258 s for each captured profile, the cutoff (0.2 of the Nyquist frequency) was chosen to process experimental noise with the Butterworth function in MATLAB. Filtering with time was performed at fixed locations, to minimize positional effects of smoothing on the observed concentration profiles. An example of a time-filtered concentration profile on the anodic side of the cell is shown in figure S10.

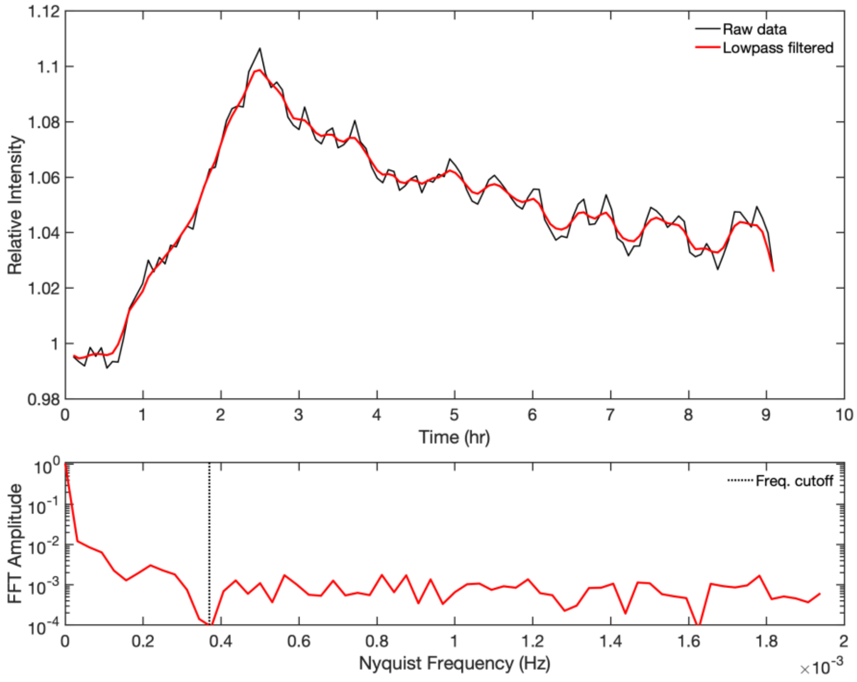

Figure S10. Example of low-pass-filtered timeseries composition data for MRI raw profiles at a fixed location.

2 molar polarization data

To demonstrate validity of the model and parameters for predicting microscopic states across wider ranges of concentration and current density, a repeat experiment was performed with 2 M $LiPF_6$:EMC, using a 2 hour polarization at a current density of 1.9 mA/cm^2, followed by an open circuit relaxation.

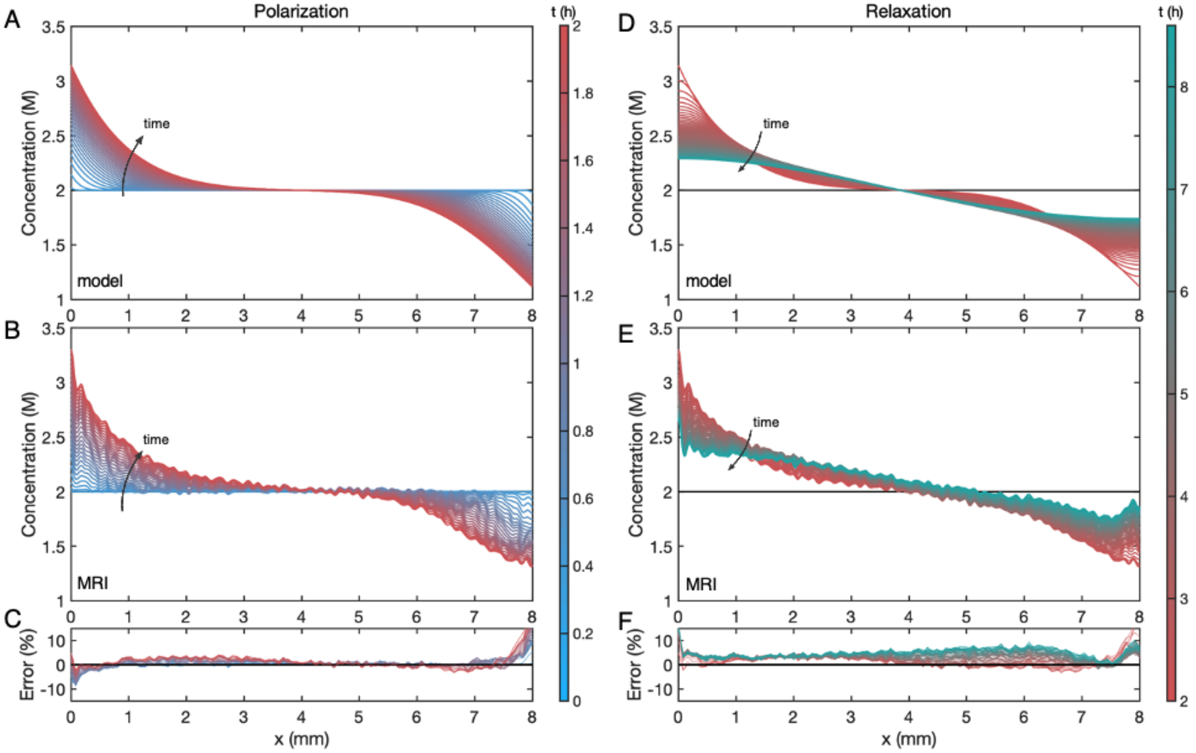

Figure S11. 2 M $LiPF_6$:EMC Comparison between dynamic concentration gradients simulated via ex-situ parameterized model and profiles captured by in-situ ^{19}F MRI during electrolyte polarization and relaxation at 25 °C. Color corresponds to the timestamp of each profile relative to the overall experiment duration.
(A) Model-simulated concentration profiles for a 2-hour pulse polarization at 1.9 mA/cm^2
(B) ^{19}F MRI concentration profiles measured for a 2-hour pulse polarization at 1.9 mA/cm^2
(C) Percentage error in microscopic concentration between MRI measurements and model predictions during the polarization step
(D) Model-simulated concentration profiles during open-circuit relaxation after the 2-hour pulse
(E) ^{19}F MRI concentration profiles measured during open-circuit relaxation after the 2-hour pulse
(F) Percentage error in microscopic states between MRI measurements and model predictions during the relaxation step

G. Electrochemical response and surface kinetics analysis

Butler–Volmer kinetics fitting

As discussed in the main article, a full characterization of the bulk transport and thermodynamic properties for super-concentrated $LiPF_6$:EMC allows the combined surface overpotentials (η_s) to be isolated by subtracting the calculated Ohmic drop (η_{ohm}) and concentration overpotential (η_c) from the measured symmetric-cell voltage (V):

$$V = \eta_s + \eta_{\mathrm{ohm}} + \eta_c. \tag{S24}$$

Here η_{ohm} and η_c may be calculated by integrating across the bulk solution phase in equation S8:

$$\eta_{\mathrm{ohm}} + \eta_c = \frac{I(t)}{A}\int_L^0 \frac{dx}{\kappa} - \frac{2RT}{F}\int_{c(t,L)}^{c(t,0)} \frac{\chi(1-t_+^0)}{c\left[1+(2\bar{V}_0-\bar{V}_{\mathrm{e}})c\right]}dc. \tag{S25}$$

The total surface overpotential for a symmetric Li-Li cell can be written as $\eta_s = \eta_+ + \eta_-$, using the surface overpotential for the lithium plating and stripping half reactions.[7] For a single lithium plating reaction at the cathode, the concentration-dependent Butler-Volmer equation with a surface concentration (c_+^{surf}), reference concentration (c_{ref}), symmetry factory (β), and surface overpotential (η_+) is:[13]

$$i = i_{0,\mathrm{ref}}\left(\frac{c_+^{\mathrm{surf}}}{c_{\mathrm{ref}}}\right)^{1-\beta}\left[\exp\left(\frac{(1-\beta)F}{RT}\eta_+\right) - \exp\left(\frac{-\beta F}{RT}\eta_+\right)\right], \tag{S26}$$

where i is the applied current density and $i_{0,\mathrm{ref}}$ is the exchange current density, the latter defined with k_a and k_c representing the anodic and cathodic reaction rate constants as

$$i_{0,\mathrm{ref}} = F k_c^{1-\beta} k_a^{\beta} c_{\mathrm{ref}}^{1-\beta}. \tag{S27}$$

Assuming β=0.5, the analytical expression may be simplified to

$$i = 2 i_{0,\mathrm{ref}} \sqrt{\frac{c_+^{\mathrm{surf}}}{c_{\mathrm{ref}}}} \sinh\left(\frac{F\eta_+}{2RT}\right). \tag{S28}$$

This can then be inverted to isolate the kinetic overpotential, as

$$\eta_+ = \frac{2RT}{F} \sinh^{-1}\left(\frac{i}{2 i_{0,\mathrm{ref}}} \sqrt{\frac{c_{\mathrm{ref}}}{c_+^{\mathrm{surf}}}}\right). \tag{S29}$$

Since hyperbolic sine is an odd function, and since two lithium/electrolyte interfaces are present in the cell, the total surface overpotential ($\eta_s = \eta_+ + \eta_-$)during polarization is

$$\eta_s = \frac{2RT}{F} \left(\sinh^{-1}\left(\frac{i}{2 i_{0,\mathrm{ref}}} \sqrt{\frac{c_{\mathrm{ref}}}{c_+^{\mathrm{surf}}}}\right) + \sinh^{-1}\left(\frac{i}{2 i_{0,\mathrm{ref}}} \sqrt{\frac{c_{\mathrm{ref}}}{c_-^{\mathrm{surf}}}}\right)\right). \tag{S30}$$

From figures 3 and 4, η_s and surface cation concentrations are known and plotted below in figure S12. Equation S30 can then be used to fit data in figure S12 (with c_{ref} = 3 M) to yield the effective exchange current density $i_{0,\mathrm{ref}}$ during the polarization, as plotted in figure S13.

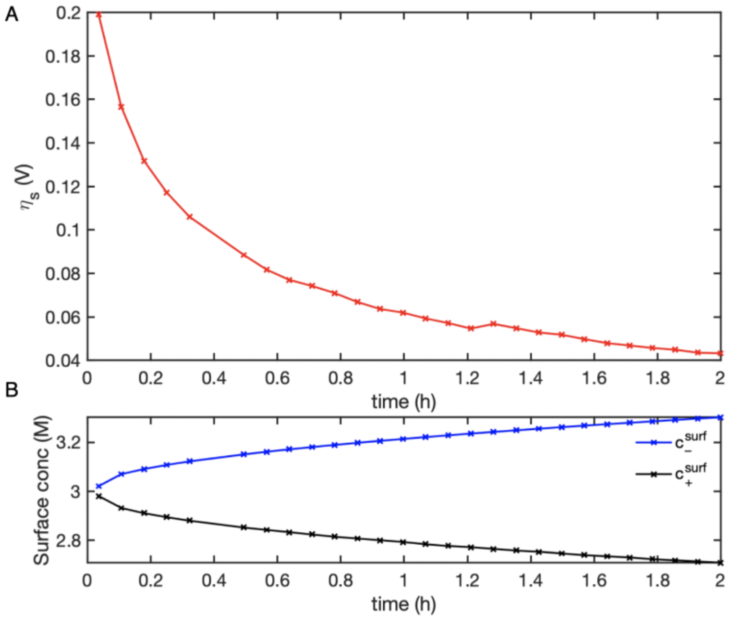

Figure S12. (A) Surface overpotential η_s during the polarization step. (B) Lithium-ion concentration in solution at the anodic surface (blue) and cathodic surface (black).

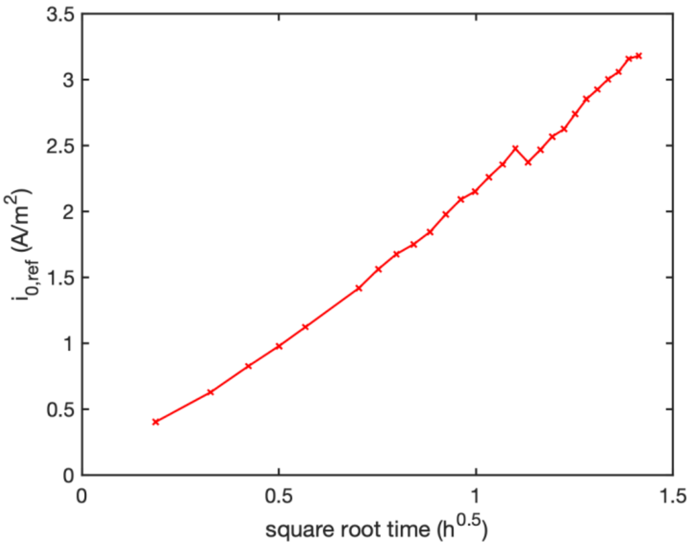

Figure S13. The effective exchange current density $i_{0,\mathrm{ref}}$ plotted with square root of polarization time, derived from fitting with equation S30.

As can be seen in figure S13, the exchange current density during the experiment increases relatively linearly with the square root of time, suggesting surface roughening during

electrodeposition.[14] The Butler–Volmer analysis here includes contributions from electrode kinetics and SEI growth. The evolution of interfacial conditions can have a large impact on electrolyte characterization experiments.[15] In the present case, the monotonic decrease in surface overpotential indicates that any growth in interfacial resistance is outcompeted by the increase in surface area from both lithium deposition and SEI formation during the plating process.

H. Dynamic Viscosities

Viscosities were measured for $LiPF_6$:EMC at temperatures of 20.0, 25.0, and 30.0 °C with a rolling-ball viscometer (Lovis 2000, Anton Paar) with 1.59 and 1.8 mm capillary diameters.

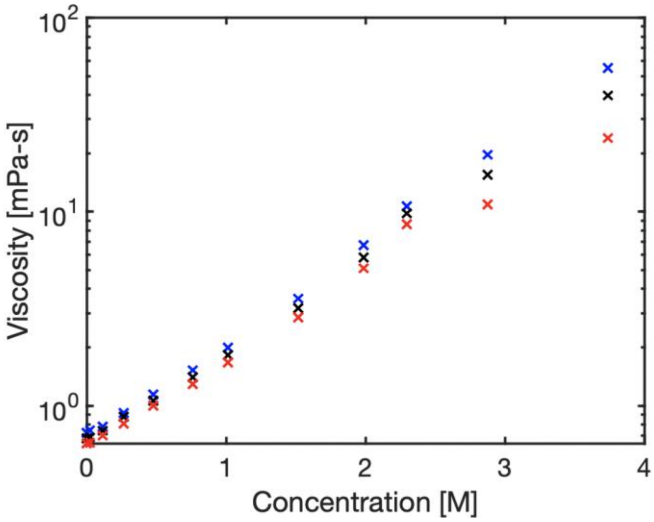

Figure S14. Semilog plot of dynamic viscosity measured across the molarity range for $LiPF_6$:EMC at 20.0 °C (blue x), 25.0 °C (black x), and 30.0 °C (red x).

I. Other Raw data

Transport and thermodynamic property measurements for $LiPF_6$:EMC were extended from the range studied previously by Wang et al.[1] up to saturation, following the experimental techniques discussed in detail by Hou and Monroe, as well as Wang et al.[1,2] Below we summarize the additional raw data that underpin this study's reported property parameterization.

Density

Table S3. Composition calibration measurements for $LiPF_6$:EMC solutions. Recorded under an argon atmosphere in a glovebox. Temperature is controlled within 0.02 °C.

ω	$\rho_{25°C}$ [g/L]	$c_{25°C}$ [M]
0.33084	1291.6	2.81
0.34517	1298.7	2.95
0.37359	1326.3	3.26
0.39210	1339.7	3.46
0.41879	1353.9	3.73
0.44633	1352.4	—
0.46776	1355.7	—
0.49981	1351.4	—
0.56003	1350.8	—
0.65174	1353.1	—

Ionic Conductivity

Table S4. Ionic conductivity measurements for $LiPF_6$:EMC solutions.

$\rho_{20\,°C}$ [g/L]	$\kappa_{25\,°C}$ [mS/cm]
1259.4	4.551
1293.5	3.449
1329.0	2.256
1360.6	1.741

Concentration cells

Table S5. Liquid-junction potentials in concentration cells for $LiPF_6$:EMC at 25 °C.

$\rho^{A}_{25\,°C}$ [g/L]	$\rho^{B}_{25\,°C}$ [g/L]	U [mV]	Std. Dev. [mV]
1268.1	1285.4	20.75	±0.16
1268.1	1299.8	37.79	±0.13
1285.4	1299.8	16.87	±0.63
1285.4	1317.0	38.41	±0.26
1299.8	1317.0	23.67	±0.07
1299.8	1333.6	43.61	±0.20
1317.0	1333.6	28.76	±0.68
1317.0	1361.4	61.83	±0.43
1333.6	1361.4	59.73	±0.16

Hittorf cells

Table S6. Hittorf-cell measurements for $LiPF_6$:EMC solutions

$\rho^{initial}_{20°C}$ [g/L]	$I_{\mathrm{pulse}}T_{\mathrm{pulse}}$ [C]	$\rho^{anodic}_{20°C}$ [g/L]	$\rho^{cathodic}_{20°C}$ [g/L]
1298.1	7.1931	1297.1	1299.2
1298.1	7.1931	1297	1299.2
1298.2	7.17936	1297.1	1299.3
1340.3	7.1141	1339.1	1341.4

Restricted diffusion

Table S7. Restricted diffusion measurements for $LiPF_6$:EMC solutions

$\rho_{20\,°C}$ [g/L]	slope fit [s^{-1}]	D_{eff} [cm^2/s]	κ_{eff} [mS/cm]	*b*
1296.9	1.15E-04	4.68E-07	2.27	2.19
1296.9	1.27E-04	5.15E-07	2.65	1.25
1296.9	1.02E-04	4.15E-07	2.51	1.57
1296.9	1.50E-04	6.06E-07	2.84	0.82
1296.9	9.25E-05	3.75E-07	1.75	3.80
1296.9	9.45E-05	3.83E-07	2.09	2.72
1328.8	1.23E-04	5.00E-07	2.03	0.89
1328.8	9.07E-05	3.68E-07	2.34	0.12
1328.8	1.26E-04	5.10E-07	2.34	0.12
1328.8	1.21E-04	4.89E-07	2.16	0.51
1328.8	1.13E-04	4.58E-07	2.34	0.12
1328.8	1.03E-04	4.16E-07	2.21	0.36
1328.8	6.42E-05	2.60E-07	1.61	2.30
1328.8	1.25E-04	5.05E-07	2.31	0.10
1328.8	8.29E-05	3.36E-07	1.99	0.99
1358.0	9.25E-05	3.75E-07	1.32	1.77
1358.0	8.85E-05	3.59E-07	1.76	0.12
1358.0	8.91E-05	3.61E-07	1.61	0.56
1358.0	6.91E-05	2.80E-07	1.54	0.82
1358.0	7.21E-05	2.92E-07	1.54	0.82

J. References

(1) Wang, A.; Hou, T.; Karanjavala, M.; Monroe, C. Shifting-Reference Concentration Cells to Refine Composition-Dependent Transport Characterization of Binary Lithium-Ion Electrolytes. *Electrochim. Acta* **2020**, *358*, 136688. https://doi.org/10.1016/j.electacta.2020.136688.

(2) Hou, T.; Monroe, C. W. Composition-Dependent Thermodynamic and Mass-Transport Characterization of Lithium Hexafluorophosphate in Propylene Carbonate. *Electrochim. Acta* **2020**, *332*. https://doi.org/10.1016/j.electacta.2019.135085.

(3) Newman, J.; Chapman, T. W. Restricted Diffusion in Binary Solutions. *AIChE J.* **1973**, *19* (2), 343–348. https://doi.org/10.1002/aic.690190220.

(4) Ehrl, A.; Landesfeind, J.; Wall, W. A.; Gasteiger, H. A. Determination of Transport Parameters in Liquid Binary Lithium Ion Battery Electrolytes. *J. Electrochem. Soc.* **2017**, *164* (4), A826–A836. https://doi.org/10.1149/2.1131704jes.

(5) Klett, M.; Giesecke, M.; Nyman, A.; Hallberg, F.; Lindström, R. W.; Lindbergh, G.; Furó, I. Quantifying Mass Transport during Polarization in a Li Ion Battery Electrolyte by in

Situ 7Li NMR Imaging. *J. Am. Chem. Soc.* **2012**, *134* (36), 14654–14657. https://doi.org/10.1021/ja305461j.
(6) Klamor, S.; Zick, K.; Oerther, T.; Schappacher, F. M.; Winter, M.; Brunklaus, G. 7Li in Situ 1D NMR Imaging of a Lithium Ion Battery. *Phys. Chem. Chem. Phys.* **2015**, *17* (6), 4458–4465. https://doi.org/10.1039/c4cp05021e.
(7) Newman, J.; Thomas-Alyea, K. E. *Electrochemical Systems*, 3rd, illustr ed.; Electrochemical Society series; John Wiley & Sons, 2004, 2004.
(8) Fong, K. D.; Self, J.; McCloskey, B. D.; Persson, K. A. Ion Correlations and Their Impact on Transport in Polymer-Based Electrolytes. *Macromolecules* **2021**, *54* (6), 2575–2591. https://doi.org/10.1021/acs.macromol.0c02545.
(9) Cabo-Fernandez, L.; Neale, A. R.; Braga, F.; Sazanovich, I. V.; Kostecki, R.; Hardwick, L. J. Kerr Gated Raman Spectroscopy of LiPF6 Salt and LiPF6-Based Organic Carbonate Electrolyte for Li-Ion Batteries. *Phys. Chem. Chem. Phys.* **2019**, *21* (43), 23833–23842. https://doi.org/10.1039/c9cp02430a.
(10) Winter, M.; Beltrop, K.; Heckmann, A.; Brunklaus, G.; Thienenkamp, J.; Placke, T. Towards High-Performance Dual-Graphite Batteries Using Highly Concentrated Organic Electrolytes. *Electrochim. Acta* **2017**, *260*, 514–525. https://doi.org/10.1016/j.electacta.2017.12.099.
(11) Wood, K. N.; Kazyak, E.; Chadwick, A. F.; Chen, K. H.; Zhang, J. G.; Thornton, K.; Dasgupta, N. P. Dendrites and Pits: Untangling the Complex Behavior of Lithium Metal Anodes through Operando Video Microscopy. *ACS Cent. Sci.* **2016**, *2* (11), 790–801. https://doi.org/10.1021/acscentsci.6b00260.
(12) Ilott, A. J.; Mohammadi, M.; Chang, H. J.; Grey, C. P.; Jerschow, A. Real-Time 3D Imaging of Microstructure Growth in Battery Cells Using Indirect MRI. *Proc. Natl. Acad. Sci. U. S. A.* **2016**, *113* (39), 10779–10784. https://doi.org/10.1073/pnas.1607903113.
(13) Monroe, C.; Newman, J. Dendrite Growth in Lithium/Polymer Systems. *J. Electrochem. Soc.* **2003**, *150* (10), A1377. https://doi.org/10.1149/1.1606686.
(14) Kushima, A.; So, K. P.; Su, C.; Bai, P.; Kuriyama, N.; Maebashi, T.; Fujiwara, Y.; Bazant, M. Z.; Li, J. Liquid Cell Transmission Electron Microscopy Observation of Lithium Metal Growth and Dissolution: Root Growth, Dead Lithium and Lithium Flotsams. *Nano Energy* **2017**, *32* (November 2016), 271–279. https://doi.org/10.1016/j.nanoen.2016.12.001.
(15) Bergstrom, H. K.; Fong, K. D.; Mccloskey, B. D. Interfacial Effects on Transport Coefficient Measurements in Li-Ion Battery Electrolytes. *J. Electrochem. Soc.* **2021**. https://doi.org/10.1149/1945-7111/ac0994.
